# Unique insights from ClinicalTrials.gov by mining protein mutations and RSids in addition to applying the Human Phenotype Ontology

**DOI:** 10.1371/journal.pone.0233438

**Published:** 2020-05-27

**Authors:** Shray Alag

**Affiliations:** The Harker School, San Jose, CA, United States of America; German Cancer Research Center (DKFZ), GERMANY

## Abstract

Researchers and clinicians face a significant challenge in keeping up-to-date with the rapid rate of new associations between genetic mutations and diseases. To remedy this problem, this research mined the ClinicalTrials.gov corpus to extract relevant biological insights, produce unique reports to summarize findings, and make the meta-data available via APIs. An automated text-analysis pipeline performed the following features: parsing the ClinicalTrials.gov files, extracting and analyzing mutations from the corpus, mapping clinical trials to Human Phenotype Ontology (HPO), and finding associations between clinical trials and HPO nodes. Unique reports were created for each mutation (SNPs and protein mutations) mentioned in the corpus, as well as for each clinical trial that references a mutation. These reports, which have been run over multiple time points, along with APIs to access meta-data, are freely available at http://snpminertrials.com. Additionally, HPO was used to normalize disease terms and associate clinical trials with relevant genes. The creation of the pipeline and reports, the association of clinical trials with HPO terms, and the insights, public repository, and APIs produced are all novel in this work. The freely-available resources present relevant biological information and novel insights between biomedical entities in a robust and accessible manner, mitigating the challenge of being informed about new associations between mutations, genes, and diseases.

## Introduction

The rapid decrease in the cost of Next-Gen Sequencing (NGS) over the past decade has led to a multitude of new NGS-based studies. Frequently, these studies associate genomic mutations—such as protein mutations and Single Nucleotide Polymorphisms [1] (SNPs)—with genes, drugs, diseases, and other phenotypes [2]. Knowledge about new associations is crucial for researchers and clinicians since understanding an individual’s genetic mutations can help identify disease risk, improve prognosis, and tailor personalized treatments [3][4]. It is currently cumbersome to keep up with the rapid rate of discoveries; however, since manual efforts to curate the literature are highly time-consuming.

ClinicalTrials.gov, run by the United States National Library of Medicine, contains more than 330,000 text documents detailing both past and present clinical trials globally [5]. A proportion of these trials includes information on SNPs, protein mutations, and genes.

Many previous researchers have effectively mined the clinical trials corpus to gain new insights: Zhang et al. 2019 [6] maps Laboratory Observation Identifier Names and Codes (LOINC [7]) to Human Phenotype Ontology (HPO [8]) terms; Gandy et al. 2017 [9] develop CTMine, which uses regular expressions for gene names to search clinical trials; Xu et al. 2016 [10] curates genetic alterations in cancer clinical trials; Su and Sanger, 2017 [11] mine ClinicalTrials.gov to develop a novel method of drug repositioning; Pradhan et al. 2018 [12] conduct a meta-analysis by automatically extracting data from ClinicalTrials.gov; and Sfakianaki et al. 2015 [13] use a Natural Language Processing (NLP) framework to mine ClinicalTrials.gov.

However, despite these important advances, mapping clinical trials to HPO terms, extracting protein mutations and SNPs [14] across the ClinicalTrials.gov corpus, and creating mutation-specific and clinical-trials-specific reports remain feats not yet accomplished.

This study analyzes ClinicalTrials.gov with six specific goals:

1. Develop a Natural Language Processing based pipeline that extracts **SNPs** and **protein mutations** instances from free text, maps their clinical trial annotations to standardized biological terms using HPO and MeSH [15] ontologies, and analyzes the complete ClinicalTrials.gov corpus to extract new insights between mutations and diseases in the clinical trials literature.
2. Generate unique reports, made freely available online, for each of the extracted **mutations**. These reports should contain the context in which the mutation is mentioned across all clinical trials, along with the associated HPO disease terms. Further, HPO annotations [16] should be used to reference other genes associated with that disease. Reports should additionally be hyper-linked to key resources for easy access to relevant content. These reports enable the presentation of new biological information in a robust and accessible manner.
3. Generate reports for each **clinical trial** that mentions a mutation. Statistics on the frequency and clinical trial categories in which mutations occur should also be provided.
4. Create a freely-available **public repository** with data associating mutations, clinical trials, disease, HPO terms, and MeSH terms. Develop APIs to access the data programmatically.
5. Repeat the analysis over **multiple time frames**, enabling future meta-analyses that may provide additional insights into mutation-disease associations over a period of time.
6. Demonstrate via an example of how the meta-data extracted from this work can be used for **machine learning.**

It is hypothesized that creating a public repository of associations between clinical trials, disease terms, SNPs, and protein mutations—and making such a repository freely-available via HTML reports, processed data, and APIs—will enable researchers and clinicians to stay up-to-date.

## Materials and methods

Two publicly-available datasets were used in this study: ClinicalTrials.gov and HPO. The methods described here are also publicly-available at protocols.io (dx.doi.org/10.17504/protocols.io.bfacjiaw).

### Datasets

#### ClinicalTrials.gov [5]

The complete repository of clinical trials displayed at ClinicalTrials.gov is available in XML format with a well-defined schema. However, analyzing clinical trial text to derive valuable insights is still a challenge as it involves parsing free-text [17].

#### HPO [8]

HPO is a standardized vocabulary of phenotype abnormalities that are seen in humans [8]. HPO is a product of the Monarch Initiative and one of the thirteen driver projects in the Global Alliance for Genomics and Health (GA4GH [18]) strategic roadmap. The HPO ontology files are available in the OBO [19] flat-file format and are easy to read and parse. HPO annotations provide a correlation between HPO terms and genes. There are three annotation files that contain associations between genes and phenotypes. The HPO files used in this project consisted of 14,961 HPO nodes, with 18,547 parent-child relationships between the nodes. Furthermore, 820,297 gene-phenotype annotations mapped across 4,312 unique genes and 8,947 individual HPO terms.

For each node, when applicable, the HPO ontology files contain a reference to MeSH, UMLS, and SnomedCT ontologies. For example, the HPO node “id: HP:0000003” with name “Multicystic kidney dysplasia” maps to the following four cross-ontology terms.

1. “xref: MSH:D021782”, which implies MeSH id D021782 and name “Multicystic Dysplastic Kidney.”
2. “xref: SNOMEDCT_US:204962002”, which implies SNOMEDCT id 204962002 and name: “Multicystic kidney”
3. “xref: SNOMEDCT_US:82525005”, which implies SNOMEDCT id 204962002 and name: “Multiple congenital cysts of kidney”
4. “xref: UMLS:C3714581”, which implies UMLS id C3714581 and name: “Multicystic dysplastic kidney”

#### MeSH [15]

Although the ClinicalTrials.gov XML does not contain MeSH ids, information about MeSH terms is present. The MeSH online tool [20] was used to retrieve MeSH ids from MeSH terms. MeSH ids are directly linked to HPO ids, in essence, enabling the association between MeSH terms to HPO nodes, as is discussed later in the Methods section.

### Approaches for finding mutations

#### Mutation format

The Human Genome Variation Society (HGVS) defines a format [21][22] for referencing variants. As per the specifications, all variants should be described at the DNA level, noting relations to an accepted reference sequence. Descriptions can be at the DNA-level (e.g., 123456A>T), RNA-level (e.g., 76a>u), and protein level (e.g., Lys76Asn). Ogino et al. 2009 [23] provides a good overview of mutation nomenclature used for molecular diagnostics.

#### RSids and SNPs

The Single Nucleotide Polymorphism database (dbSNP) repository [24] assigns a unique id to variations including SNPs, short nucleotide insertions and deletions, and short tandem repeats. These ids are called RSids and appear in the format rs##. For example, the RSid rs35652124 maps to the following mutations in HGVS format NC_000002.12:g.177265345T>C, NC_000002.11:g.178130073T>C [25] and is a mutation on chromosome 2 at location 177265345, with associated gene *NFE2L2*. Public repositories, such as ClinVar [26] archive human genetic variants and interpretations of mutations’ significance to diseases. Such repositories use RSids as unique identifiers. ClinVar [24], for instance, has more than 400 thousand RefSNPs.

#### SNP extraction

SNPs can be extracted with simple text processing methods as all SNPs follow the RSid format of beginning with the letters rs and having multiple numbers that follow the initial letters. For example, an SNP may be under the id rs9939609 or rs6971.

#### Protein mutation extraction

Several tools are available to mine mutations from the text. Some examples of such tools are:

1. MutationFinder [27] is a simple-to-use package that uses a rule-based approach with more than 1500 regular expressions to extract protein mutations from the text.
2. Open Mutation Miner [28] is a tool that detects and annotates protein mutations by combining rules with the MutationFinder. It also maps the impact of the mutation by integrating Gene Ontology (GO) [29].
3. SNP Extraction Tool for Human Variations (SETH) [30] is an entity recognition tool that extends MutationFinder. SETH can recognize the following subtypes of mutations: substitution, deletion, insertion, duplication, insertion-deletion (insdel), inversion, conversion, translocation, frameshift, short-sequence repeat, and literal dbSNP mention. SETH also normalizes the genetic variant to a standard RSid.
4. tmVar [31] is a mutation extraction tool based on a conditional random field model and covers a wide range of sequence variants at both protein and gene levels in HGVS format.
5. tmVar 2 [32] builds on tmVar to automatically extract and map variants to unique identifiers (dbSNP RSIDs). tmVar 2.0 achieved nearly 90% in F-measures for normalizing the mutations ids and also compared well to SETH.

Yepes and Verspoor, 2014 [33] provide an overview of relative performance between the different mutation extraction tools. For this study, the MutationFinder tool was chosen for its precision and recall. A text processing pipeline was developed to first extract RSids (SNP mutations) using pattern matching; the MutationFinder tool was then applied to extract protein mutations. No changes were made to the MutationFinder Java code.

### Programming packages

Tools used throughout the project are displayed in Table 1. Java was the primary programming language.

**Table 1:** Software libraries used in this study.

	Software	Details
1	SAX Parser [34]	Parsing XML of Clinical Trials
2	Apache OpenNLP [35]	NLP parser for SNP mutations
3	MutationFinder [27]	Protein mutation detection
4	Bootstrap [36]	CSS files for HTML
5	Amazon Web Services (AWS [37])	To host HTML reports
6	Jupyter Notebook (Google Colab [38])	Python example to access API
7	Java Client API	To access results programmatically

The software tools used and their descriptions. Software libraries 1, 2, and 3 aided in locating mutations in the text files while libraries 4 and 5 facilitated the creation of the reports and website. Software tools 6 and 7 were employed to enhance the accessibility of the results.

### Analysis steps

**Fig 1:**
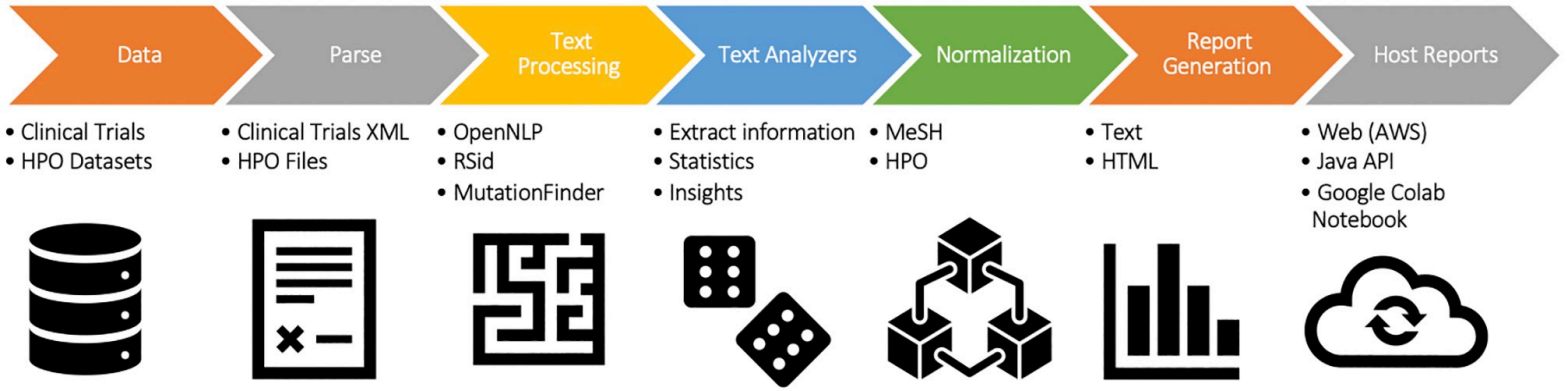
Seven steps of the pipeline. Methodology to mine ClinicalTrials.gov to extract unique insights for understanding SNPs and mutations. Each of the steps is described in detail in the “Analysis Steps” section.

The seven main analysis steps are illustrated in Fig 1 and described in detail below.

1. **Download:** XML files from ClinicalTrials.gov and HPO data files.
2. **Parse:** The Java SAX parser framework efficiently parsed the ClinicalTrials.gov XML files. In this step, for a given clinical-trial XML file, a fully-instantiated JavaBean class was created to represent the Clinical Trial. Key XML. fields used in this study include Title, Summary, Study Type, Description, Outcomes, Arm, Study Design, MeSH Terms, Outcomes, Conditions, Intervention, Phase, Observational Model, and Keywords. The MeSH terms referenced in the XML were mapped to their MeSH ids using the procedure explained below:
  - Created a list of MeSH terms referenced across all clinical trials.
  - Retrieved MeSH ids using the MeSH online tool [20] for each of the MeSH terms in the list.
  In the same manner, the HPO ontology file was parsed to create a parent-child hierarchy: HPO annotation files were parsed, and associations between HPO nodes and genes were noted.
3. **Text Processing:** The Apache OpenNLP library was utilized to parse the clinical trials into sentences. Using OpenNLP, a series of classes were created to effectively tokenize the various sentences. Regular Expressions were used to detect SNPs and protein mutations. For instance, detailed below is the process of detecting key entities:
  - Parse XML using SAX Parser.
  - Create a JavaBean instance with attributes.
  - Tokenize text by splitting the paragraphs into sentences and then sentence to tokens.
  - Regular Expressions were used to determine if a specific token was either a protein mutation or an SNP. As detailed in “SNP Extraction” and “Protein mutation,” particular regular expressions denoted the presence of a mutation.
4. **Text Analyzers:** Several crawlers were created to traverse through the local XML files and extract relevant information. Functions of the text processors are the following: create an index of all clinical trials; associate conditions with the clinical trials; extract SNPs, protein mutations, and MeSH terms from the tokens; derive frequency information and reports for SNPs, protein mutations, HPO nodes, MeSH nodes, etc.; and map clinical trials to HPO terms (in essence, normalizing to HPO nodes). Normalization is discussed further below.
5. **Normalization:** Clinical trials were mapped to HPO nodes through the following process:
  - MeSH ids were associated with HPO ids using the HPO data file.
  - HPO ids are linked to an HPO node. Thus, clinical trials were correlated to MeSH terms, MeSH ids, and finally HPO nodes.
  The steps normalized the HPO terms to standardize correlations between overlapping terms.
6. **Report Generators:** Reports were generated to analyze the processed data, display detailed information for each of the mutations, and showcase elements of the clinical trials in which the mutations appear.
7. **Host Reports:** The final reports are hosted on an AWS S3 bucket [37]. Note that these static-hyperlinked-HTML reports support user interactions. Java client APIs, along with a Google Colab document (Jupyter Notebook using Python), was created to make the produced analytics and results accessible programmatically.

### Machine learning example

We conducted a simple example of how the insights produced from this work can be applied biologically via machine learning. In this instance, clusters of similar HPO terms are desired for research purposes. It was decided to identify alike HPO terms by analyzing the correlations between SNPs and HPO terms. For example, if two HPO terms were linked to an SNP, those two terms would have a high probability of being related. The Java code for this example is available at the SNP Miner Trials homepage (package: com.snpminertrials.ct.snp.ml.HPOSnpClustering). To illustrate how machine learning can be applied to the results and analytics produced, the following procedure was applied to solve the presented example:

1. Use the tabular data (available from the homepage) to create an incidence matrix where each row is an HPO node and each column an SNP. There are *m* HPO nodes and *n* SNPs. A value of one is inputted every time the *m*_*i*_ HPO node is correlated with the *n*_*i*_ SNP term. Else, a value of zero is inserted for the element.
2. Normalize the data by creating a unit vector for each HPO term. Unit vectors are obtained by dividing each element of a row by the magnitude of that row.
3. For each HPO term, compute the pair-wise dot product between its vector and all other vectors. The resulting vector is a metric of normalized correlation.
4. Sort the results to create a prioritized list of related HPO terms

Hierarchical Clustering [41] or K-Means [42] could also be used to find clusters of related HPO terms. A similar process can be used with protein mutations—in place of SNPs—as well. Alternatively, HPO terms could be clustered based on both protein and SNP mutations. The rows and columns can be switched to cluster similar SNP/protein mutations by their associated HPO terms [43].

## Results

The “Results” section comprises of six sub-topics:

1. Details on the created public repository to provide access to the data used, reports created, correlations mapped, and APIs produced.
2. Insights about the ClinicalTrials.gov corpus after normalizing the data using MeSH and HPO ontologies.
3. Insights about the mined SNPs.
4. Insights about the extracted protein mutations.
5. Analysis of popular interventions.
6. Findings related to the machine learning example.

### Public repository

#### Web page to access longitudinal analysis data, reports, and APIs

All analysis results are accessible via the SNP Miner Results home page, available at http://snpminertrials.com. A view of the home page is seen in S1 Fig. The web page provides access to data and reports from multiple time frames. As of March 2020, there are two analysis time points: August 2019 and March 2020. Additionally, the home page has links to Java APIs and Google Colab pages, which facilitate easy local access to the insights and results of this research. The SNP Miner Results home page provides the latest analysis results, and—due to the constant influx of new clinical trials, enhancements to HPO, and HPO annotation files—the results are subject to change.

Java APIs, as well as a Google Colab Notebook (see S1 Fig) with Python, allow the results to be easily accessed programmatically.

The functionalities of the various APIs are to retrieve information about the following:

1. The MeSH terms and MeSH ids used to tag the Clinicaltrial.gov corpus
2. HPO terms and their corresponding clinical trials
3. RSids and their corresponding clinical trials
*Relevant MeSH ids and their correlated clinical trials
*Relevant HPO ids and their correlated clinical trials
6. Protein mutations and their corresponding clinical trials

*Only the specific terms that have any correlation to a mutation are shown.

Additionally, there are results discussing the machine learning example mentioned earlier.

### Term normalization

The clinical trial XML contains a field called “Condition”, which is a free-formed annotation associated with the clinical trial. S2 Fig shows frequently occurring conditions (referenced more than 1,000 times) across the clinical trial documents. Since these conditions are free-formed and not mapped to a standard ontology, multiple distinct terms refer to the same condition. For example, six terms that refer to “Type 1 Diabetes”—“Diabetes Mellitus, Type 1,” “Type 1 Diabetes,” “Type 1 Diabetes Mellitus,” “Type1diabetes,” “Type1 Diabetes Mellitus,” and “Diabetes Mellitus Type 1” appear throughout the clinical trials. Standard ontologies such as MeSH and HPO map these variant terms to a single ontology node: D003922 [39] for MeSH and HP:0100651 [40] for HPO. There were 87,656 unique conditions, and 559,918 total condition mentions. Thus, normalization was pivotal in standardizing the results.

In the XML data, each clinical trial contains a list of associated MeSH tags. As described in the “Methods” section, these MeSH tags were useful in linking MeSH terms to HPO terms and MeSH ids to HPO ids.

Using information about MeSH tags, multiple analytics were produced: 6,643 unique MeSH tags have been cited 568,784 times across the 332,418 clinical trials; approximately 81% of the clinical trials have a MeSH annotation, and around 62% of the trials have a MeSH annotation with an associated HPO term mapped to a gene. S2 Fig displays all of the MeSH terms with at least 2,000 total tags ranked by frequency.

### Results from extracting RSids

There were 566 unique RSids across 368 clinical trials, with a total of 798 mentions. Table 2 contains the top three most frequently occurring RSids, while S2 Fig shows a tabular view of frequently occurring SNPs and HPO terms. rs12979860 co-occurs with “HP:0012115 Hepatitis” 33 times. rs12979860, which occurs near *IL28B*, is in fact used for selecting Hepatitis C treatment [44], validating the methodology and results. Other notable SNPs referenced multiple times across the corpus are rs6971, which appears is associated with brain diseases [46] and rs9939609, which is associated with fat mass and obesity [47]. All of these results help validate the pipeline employed since all of these SNPs have already been commonly known and studied.

**Table 2:** Most frequent RSids across ClinicalTrials.gov.

	RSid	Count	HPO Node	HPO Node Name	Count
1	rs12979860	38	HP:0012115	Hepatitis	33
			HP:0200123	Chronic hepatitis	2
			HP:0001402	Hepatocellular carcinoma	2
			HP:0030731	Carcinoma	1
			HP:0001392	Abnormality of the liver	1
2	rs6971	26	HP:0002511	Alzheimer disease	4
			HP:0006802	Abnormal anterior horn cell morphology	2
			HP:0007354	Amyotrophic lateral sclerosis	2
			HP:0100753	Schizophrenia	1
			HP:0000729	Psychosis	1
			HP:0000709	Encephalitis	1
			HP:0002383	Psychosis	1
			HP:0000717	Autism	1
			HP:0000716	Depressivity	1
			HP:0002180	Neurodegeneration	1
			HP:0001658	Myocardial infarction	1
			HP:0001268	Mental deterioration	1
3	rs9939609	11	HP:0001513	Obesity	3
			HP:0000819	Diabetes mellitus	1
			HP:0001824	Weight loss	1
			HP:0000855	Insulin resistance	1
			HP:0100651	Type I diabetes mellitus	1

Most frequent RSids extracted across ClinicalTrials.gov.

#### Validation case

To further validate the pipeline, 37 SNPs associated with “HP:0003002 Breast carcinoma” were analyzed. These SNPs are rs1011970, rs10407022, rs1045485, rs10941679, rs10995190, rs11045585, rs11133360, rs11249433, rs12762549, rs13281615, rs13387042, rs16942, rs1800566, rs2002555, rs2046210, rs2237060, rs2241193, rs2297480, rs236114, rs2380205, rs271924, rs2981582, rs3803662, rs3817198, rs4073, rs4646, rs4973768, rs614367, rs6504950, rs704010, rs7333181, rs7349683, rs889312, rs909253, rs9344, rs9457827, and rs999737. Each one of these were manually verified for associations with breast cancer. As expected, each and every one of them had a known association with breast cancer, further illustrating the accuracy and effectiveness of the methodology. The Java API toolkit includes an API that returns a list of SNPs for an associated HPO node.

#### MeSH terms, HPO terms, and reports

S2 Fig illustrates the most prominent MeSH ids referenced across the 368 clinical trials with RSids. Interestingly, the first set of MeSH terms was related to Hepatitis, with more than 10% (37 out of 368) of clinical trials falling into this category, demonstrating the quantity of research involving mutations and Hepatitis.

The most cited HPO terms fall into the areas of Hepatitis, Diabetes, Cancer (Breast carcinoma, Leukemia), abnormality of the cardiovascular system, and Schizophrenia. S2 Fig shows the key HPO terms with associated SNPs across the clinical trial corpus. The 368 clinical trials mapped to 136 different HPO terms and were referenced 368 times. The frequency of HPO terms sheds light on the areas that researchers are prominently interested in.

Table 3 shows the top HPO nodes with the highest occurring RSids. Breast carcinoma had 38 unique RSids associated with it, suggesting that genetic mutations possibly influence Breast Cancer. Other diseases with the most number of associated RSids include Impulsivity, Aggressive behavior, Diabetes mellitus, Hepatitis, and Asthma.

**Table 3:** HPO Terms with the most number of associated RSids.

	HPO Id	Name	#	RSid
1	HP:0003002	Breast carcinoma	37	rs1011970,rs10407022,rs1045485,rs10941679,rs10995190,rs11045585,rs11133360,rs11249433,rs12762549,rs13281615,rs13387042,rs16942,rs1800566,rs2002555,rs2046210,rs2237060,rs2241193,rs2297480,rs236114,rs2380205,rs271924,rs2981582,rs3803662,rs3817198,rs4073,rs4646,rs4973768,rs614367,rs6504950,rs704010,rs7333181,rs7349683,rs889312,rs909253,rs9344,rs9457827,rs999737,
2	HP:0100710	Impulsivity	23	rs1042713,rs1079598,rs1150226,rs1549339,rs16111115,rs1672717,rs1800497,rs1800955,rs1801253,rs2242447,rs2278392,rs2550946,rs4532,rs4680,rs4994,rs518147,rs553668,rs5569,rs6269,rs6280,rs6295,rs6296,rs6311
3	HP:0000718	Aggressive behavior	23	rs1042713,rs1079598,rs1150226,rs1549339,rs16111115,rs1672717,rs1800497,rs1800955,rs1801253,rs2242447,rs2278392,rs2550946,rs4532,rs4680,rs4994,rs518147,rs553668,rs5569,rs6269,rs6280,rs6295,rs6296,rs6311
4	HP:0000819	Diabetes mellitus	22	rs10830963,rs12469968,rs13266634,rs2266782,rs2281135,rs2284872,rs2294918,rs35652124,rs35874116,rs35874116rs,rs3765467,rs3788979,rs5215,rs5219,rs738409,rs7565794,rs780094,rs780094s,rs78408340,rs7903146,rs9701796,rs9939609
5	HP:0012115 HP:0200123	Hepatitis Chronic hepatitis	20	rs10813831,rs1127354,rs11795404,rs12356193,rs12979860,rs12992677,rs17037122,rs179008,rs2066842,rs2067085,rs2464266,rs3853839,rs41308230,rs4588,rs5743844,rs6592052,rs7041,rs7270101,rs7549785,rs8099917
6	HP:0002099	Asthma	18	rs1042711,rs1042713,rs1042714,rs1042718,rs11958940,rs11959427,rs12654778,rs12936231,rs1504982,rs17778257,rs1800888,rs1801275,rs1805010,rs2053044,rs2895795,rs324011,rs324015,rs4950928
7	HP:0001257	Spasticity	18	rs1049522,rs1049524,rs137852620,rs2032892,rs2269272,rs2269273,rs2562582,rs2731886,rs377637047,rs4869675,rs4869676,rs529802001,rs544684689,rs547987105,rs549927573,rs550842646,rs562696473,rs573562920
8	HP:0001638	Cardiomyopathy	18	rs1042522,rs1042522s,rs1056892,rs10836235,rs10865801,rs1128503,rs1149222,rs13058338,rs1465952,rs1786378374,rs1883112,rs2229774,rs2279744,rs35599367,rs3761624,rs45511401,rs4673,rs7853758
9	HP:0001677	Coronary arteryatherosclerosis	16	rs10153820,rs1143623,rs1143633,rs1143634,rs12041331,rs16944,rs16969968,rs17561,rs1761667,rs2305619,rs4848306,rs6434222,rs7586970,rs7903146,rs8069645,rs8176528
10	HP:0001909	Leukemia	15	rs10509681,rs11572080,rs12459419,rs172378,rs2032582,rs230561,rs25531,rs3816527,rs396991,rs4880,rs4958351,rs6190,rs628031,rs776746,rs904627

The 368 clinical trials with RSids mapped to 136 unique HPO terms.

An HTML report was created for each of the 566 unique RSids, and reports over multiple time periods are freely available via the home page (http://www.snpminertrials.com). As shown in S1 Fig, each report contains a list of the clinical trials in which the SNP appears, along with the sentences containing the SNP. Each clinical trial report also shows the mapped HPO as well as MeSH terms, both of which are hyperlinked to other reports and external resources. As shown in S1 Fig, the HPO terms and their associated genes are also displayed at the bottom of the report. All 566 SNPs are displayed on the left-hand side of the report to enable easy navigation across the RSids.

Similarly, an HTML report was generated for each of the 368 unique clinical trials that mentioned SNPs. Reports, over multiple time periods, are freely available. As shown in S1 Fig, all reports contain the details of the clinical trial, the list of SNPs mentioned, and the sentences in which each SNP appears. Every clinical trial report shows the mapped HPO and MeSH terms, which are also hyperlinked. S1 Fig highlights the unique RSid terms and their associated sentences, which are also displayed at the bottom of the report. All the 368 clinical trial ids are displayed on the left-hand side of the report to enable easy navigation across the clinical trials.

### Results of extracting protein mutations from the clinical trial corpus using MutationFinder

There were 962 unique protein mutations across 1,939 clinical trials, with a total of 3,881 mentions.

Table 4 contains the top four most frequently occurring protein mutations. The protein L858R is cited in 293 clinical trials, out of which 233 clinical trials mapped to HPO node “HP:0030358, Non-small cell lung carcinoma,” suggesting a correlation between L858R and Lung Cancer. The 293 clinical trials that mention the L858R map to 21 HPO nodes, most of which are associated with Cancer. E.g., “HPO:0100526 Neoplasm of the lung”, “HP:0030731 Carcinoma”, “HP:0030692 Brain Neoplasm”, etc. Similarly, T790M (synonym, Thr790Met) is cited across 289 clinical trials, which frequently map to cancer-related HPO nodes, indicating the vast amount of Cancer research performed. V600E and T315I, with 228 and 98 citations respectively, are the next two most commonly cited protein mutations. V600E is associated with Cutaneous melanoma, Neoplasm of the large intestine, and Thyroid adenoma, while T315I is associated with Leukemia, Chronic myelogenous Leukemia, and Myeloid leukemia.

**Table 4:** Most frequent mutations across ClinicalTrials.gov.

	Mutation	Synonyms	Count	HPO Node	HPO Node Name	Count
1	L858R	leucine to arginine at codon 858 leucine-to-arginine mutation at codon 858	293	HP:0030358	Non-small cell lung carcinoma	233
				HP:0100526	Neoplasm of the lung	165
				HP:0030731	Carcinoma	16
				HP:0002664	Neoplasm	6
				HP:0030692	Brain neoplasm lung morphology	4
				HP:0002088	Cutaneous melanoma	2
				HP:0012056	Pleural effusion	2
				HP:0002202	14 more…	2
				…		…
2	T790M	Thr790Met	289	HP:0030358	Non-small cell lung carcinoma	222
				HP:0100526	Neoplasm of the lung	154
				HP:0030731	Carcinoma	20
				HP:0002664	Neoplasm	10
				HP:0002088	Abnormal lung morphology	4
				HP:0030357	Small cell lung carcinoma	3
				HP:0005584	Renal cell carcinoma	2
				…	17 more…	..
3	V600E		228	HP:0012056	Cutaneous melanoma	98
				HP:0100834	Neoplasm of the large intestine	31
				HP:0030358	Non-small cell lung carcinoma	28
				HP:0100526	Neoplasm of the lung	25
				HP:0002664	Neoplasm	21
				HP:0030731	Carcinoma	15
				HP:0000854	Thyroid adenoma	13
				…	53 more…	13 ..
4	T315I	Thr315Ile threonine 315 to isoleucine	98	HP:0001909	Leukemia	83
				HP:0005506	Chronic myelogenous leukemia	73
				HP:0012324	Myeloid leukemia	67
				HP:0005526	Lymphoid leukemia	23
				HP:0004808	Acute myeloid leukemia	5
				HP:0002863	Myelodysplasia	4
				…	14 more…	…

The top four commonly cited protein mutations across the clinical trials and their related HPO nodes.

**Fig 2:**
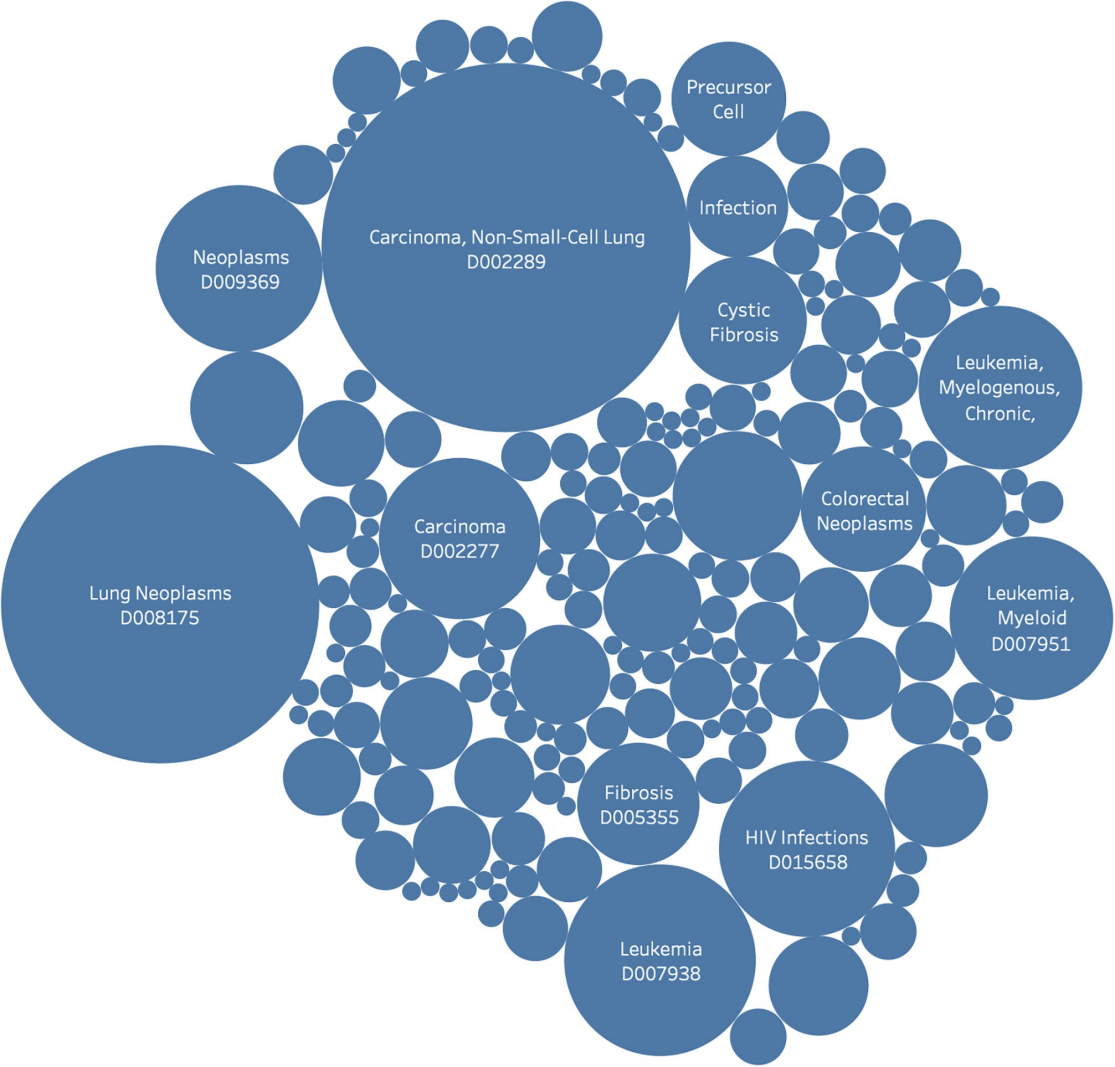
Bubble graph showing the key MeSH nodes used to tag clinical trials with protein mutations.

**Fig 3:**
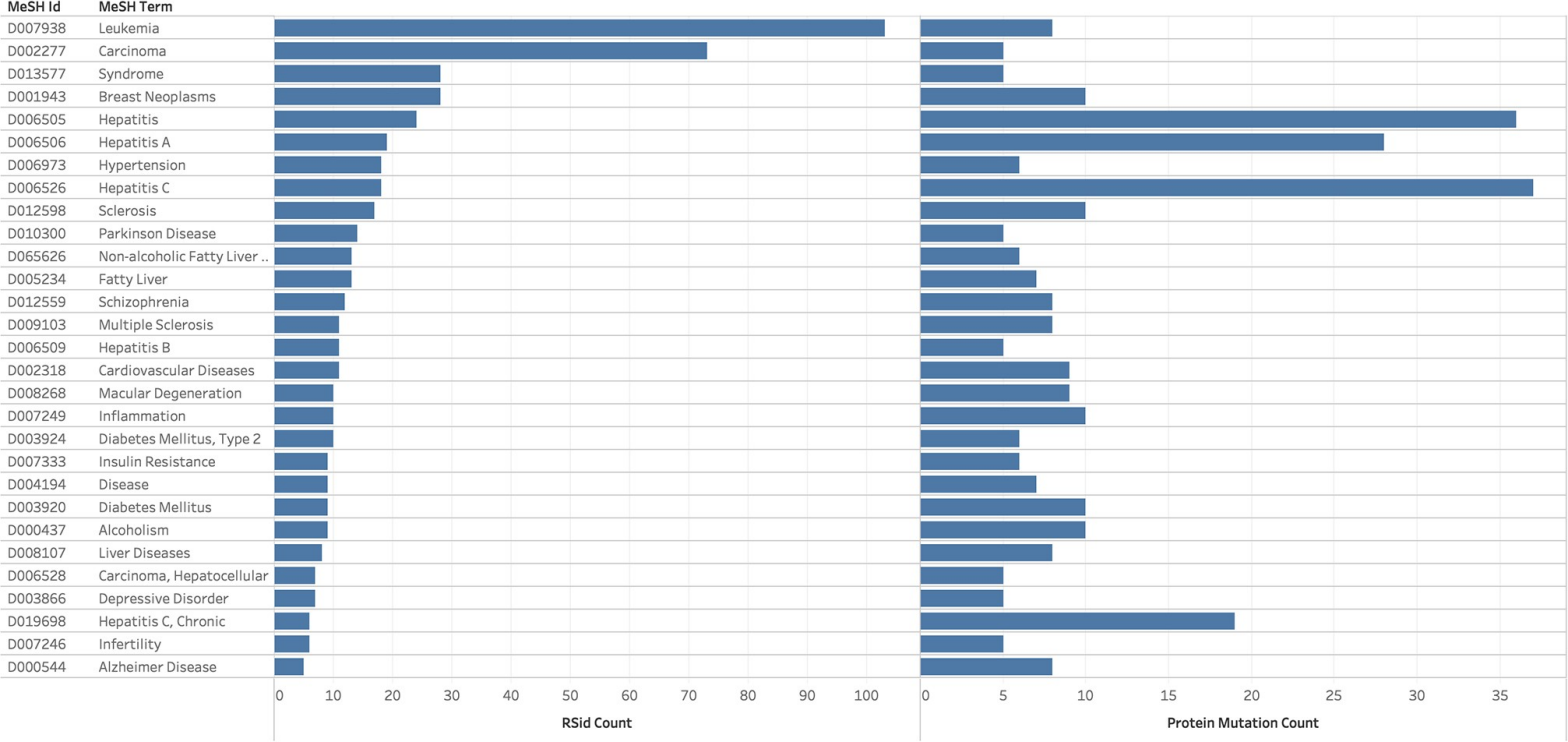
Common MeSH terms for clinical trials with RSid and protein mutation frequencies.

The 1,939 unique clinical trials that referenced protein mutations were subsequently analyzed. MeSH terms that appear frequently across clinical trials that contain protein mutations are shown in Fig 2. Fig 3 illustrates MeSH terms that frequently appear for both the RSid and protein mutation cases. In Fig 3, multiple MeSH terms are related to Hepatitis and Cancer, further demonstrating the quantity of research in these fields.

Similarly, Table 5 portrays the top HPO terms referenced across these 1,939 clinical trials with protein mutations. The HPO node HP:0030358 “Non-small cell lung carcinoma” is associated with 382 clinical trials, followed by HP:0100526 “Neoplasm of the lung” with 284 clinical trials. “Leukemia”, “Cutaneous melanoma,” “Myeloid Leukemia,” “Neoplasm,” “Chronic myelogenous leukemia,” “Myeloid leukemia,” “Carcinoma,” “Neoplasm of the large intestine,” and “Lymphoma” are the remaining HPO terms with the most number of associated clinical trials. The quantity of Cancer nodes possibly suggests a correlation between mutations and Cancer.

**Table 5:** HPO Terms with the most cited protein mutations found by MutationsFinder in ClinicalTrials.gov.

	HPO Id	Number Clinical Trials	HPO Node Name
1	HP:0030358	382	Non-small cell lung carcinoma
2	HP:0100526	284	Neoplasm of the lung
3	HP:0001909	106	Leukemia
4	HP:0012056	103	Cutaneous melanoma
5	HP:0002664	78	Neoplasm
6	HP:0005506	75	Chronic myelogenous leukemia
7	HP:0012324	75	Myeloid leukemia
8	HP:0030731	73	Carcinoma
8	HP:0100834	44	Neoplasm of the large intestine
10	HP:0002665	36	Lymphoma

The 1,939 clinical trials with mutations mapped to 332 unique HPO terms and were referenced 2,447 times.

Next, analyzing the number of protein mutations for each of the reference HPO terms provides insights, as shown in Table 6. HP:0002664 “Neoplasm” has 75 associated protein mutations, while HP:0003002 ‘Breast Carcinoma’ is next with 73 mutations. “Carcinoma”, “Lymphoma,” “Neoplasm of the lung,” “Leukemia,” “Non-small cell lung carcinoma,” and “Non-Hodgkin lymphoma” are the other top-six HPO nodes with the most number of associated protein mutations.

**Table 6:** HPO Terms with the most number of associated mutations.

	HPO Id	Name	#	Mutations
1	HP:0002664	Neoplasm	75	C10D,C377T,C677T,C797S,D816V,D835V,D842V,E10A,E17K,E542K,E545K,F1174L,F31I,G12C,G12D,G12V,G13D,G156A,G20210A,G719A,G719C,H1047R,H1112L,H1112Y,H1124D,K652E,L1213V,L265P,L858R,L861Q,M1149T,M1268T,P1009S,P13K,P1446A,P286R,P4503A,Q12H,Q21D,R132C,R132G,R132H,R132L,R132S,R132V,R140L,R140Q,R140W,R172G,R172K,R172M,R172S,R172W,R988C,T1010I,T1191I,T315I,T790M,V1110L,V1206L,V1238I,V411L,V57I,V600D,V600E,V600K,V600M,V600R,V617F,V941L,Y1248C,Y1248D,Y1248H,Y1253D,Y842C
2	HP:0003002	Breast carcinoma	73	A289T,A864V,C3435T,D538G,D769H,D769N,D769Y,D988Y,E380Q,E542K,E545K,E709K,E757A,G309A,G309E,G598V,G776C,G776V,H1047R,I655V,I767M,L536H,L536P,L536Q,L536R,L755P,L755S,L786V,L841V,L858R,L861Q,L869R,P125A,P12A,P13K,P187S,P535H,P596L,R108K,R222C,R572Y,R678Q,R831C,R831H,R849W,R896C,S310F,S310Y,S463P,S653C,S768I,S8814A,S9313A,T47D,T733I,T790M,T798I,T798M,T862I,V244M,V534E,V600E,V659E,V697L,V742I,V769M,V773M,V774M,V777L,V842I,Y537C,Y537N,Y537S
3	HP:0030731	Carcinoma	57	C3435T,C420R,C938A,E10A,E542K,E545A,E545D,E545G,E545K,G1049R,G12C,G20210A,G719A,H1047L,H1047R,H1047Y,I105V,I10A,K751Q,L8585R,L858R,L861Q,M1043I,N345K,N375S,P13K,P286R,Q12W,Q546E,Q546K,Q546L,Q546R,R399Q,R776G,R831C,R88Q,S100P,S1400A,S1400C,S1400D,S1400E,S1400F,S1400G,S1400I,S1400K,S1900A,S1900C,S1900D,S768I,T790M,V411L,V600E,V600K,V600R,V617F,V762A,V843I
4	HP:0002665	Lymphoma	52	A677G,A677V,A687V,C282Y,C481S,E571K,F1174L,G156A,G71R,H1112L,H1112Y,H1124D,H63D,I10A,I1171N,L1213V,L265P,M1149T,M1268T,P1009S,P11A,P13K,P140K,P4503A,Q12H,Q21D,Q28D,R131H,R988C,T1010I,T1191I,T315I,T351I,T790M,V1110L,V1206L,V1238I,V158F,V158M,V600E,V617F,V66M,V941L,Y1248C,Y1248D,Y1248H,Y1253D,Y641C,Y641F,Y641H,Y641N,Y641S
5	HP:0100526	Neoplasm of the lung	52	C1156Y,C797S,D594G,F1174C,F1174V,G1202R,G1269A,G12C,G12D,G469A,G719A,G719C,G719S,G776C,G776V,I10A,L1196M,L1198F,L523S,L755S,L833F,L8585R,L858R,L859R,L861G,L861Q,L861R,N375S,P13K,P4503A,R776G,R831C,S1400A,S1400C,S1400D,S1400E,S1400F,S1400G,S1400I,S1400K,S1800A,S1900A,S1900C,S1900D,S768I,T790M,T81C,T890M,V600E,V769L,V777L,V843I
6	HP:0001909	Leukemia	51	C282Y,C481S,D816V,D835Y,E255K,E255V,F317C,F317L,F317S,F317V,F31I,F359C,F359V,G250E,G71R,H369P,H63D,L248R,L248V,N682S,P140K,P1446A,P4503A,Q12H,Q252H,R132C,R132G,R132H,R132L,R132S,R132V,R140L,R140Q,R140W,R172G,R172K,R172M,R172S,R172W,S1612C,S9333A,T315A,T315I,T351I,V158M,V299L,V57I,V600E,V617F,V66M,Y253H
7	HP:0030358	Non-small cell lung carcinoma	43	C797S,C8092A,D594G,F1174C,F1174V,G1202R,G1269A,G12C,G12D,G12V,G13D,G2032R,G469A,G719A,G719C,G719S,G776C,G776V,I10A,L1196M,L523S,L755S,L833F,L8585R,L858R,L861G,L861Q,L861R,P13K,P4503A,R776G,R831C,S1800A,S1900A,S1900C,S768I,T790M,T81C,V600E,V600K,V769L,V777L,V843I
8	HP:0012539	Non-Hodgkin lymphoma	42	A1298C,A222V,A677G,A677V,A687V,C677T,F1174L,G71R,H1112L,H1112Y,H1124D,L1213V,M1149T,M1268T,P1009S,P13K,P140K,P4503A,Q12H,Q30R,R988C,T1010I,T1191I,T315I,T790M,V1110L,V1206L,V1238I,V158M,V617F,V66M,V941L,Y1248C,Y1248D,Y1248H,Y1253D,Y641C,Y641F,Y641H,Y641N,Y641S,Y93C

The 1,939 clinical trials with mutations mapped to 332 unique HPO terms and were referenced

2,447 times.

**Fig 4:**
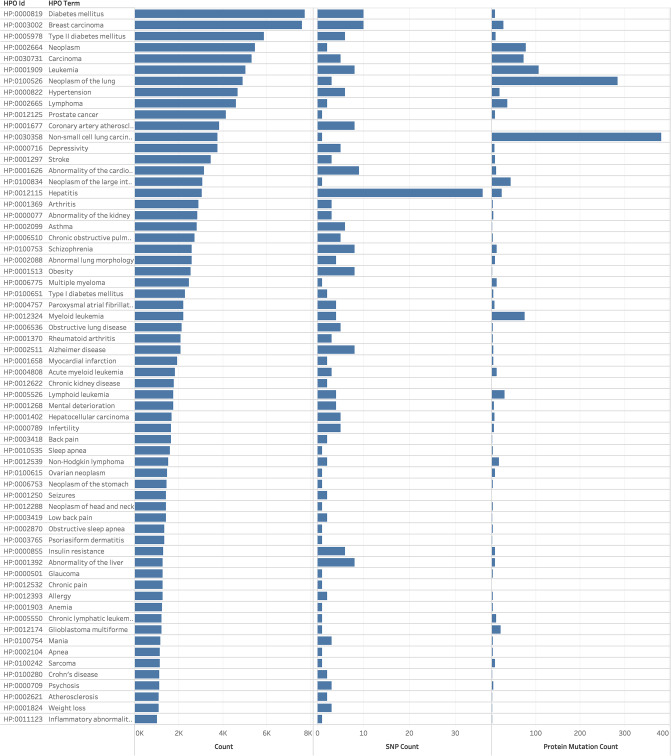
Frequency of different HPO terms across clinical trials, across trials with RSids, and across trials with protein mutations.

Fig 4 shows the distribution of HPO terms across (a) all clinical trials, (b) those with RSids, and (c) those with protein mutations. Interestingly, Diabetes Mellitus is the most commonly occurring HPO Term across all clinical trials.

HTML reports were created for each of the 962 unique protein mutations and are freely available from the SNP Miner home page (http://snpminerptrials.com). As shown in S1 Fig, each report contains a list of clinical trials where the protein mutation appears, along with the sentences containing the mutations. Each protein mutation report shows the mapped HPO as well as MeSH terms. All 962 protein mutations are displayed on the left-hand side of the report to enable easy navigation. Similarly, reports for each of the clinical trials which reference a protein mutation are also available.

### Interventions

Interventions (or treatments) are the focus of a clinical trial and are categorized into eleven different types, as shown in Table 7. There are 573,887 unique Intervention tags across the eleven different Intervention Types.

**Table 7:** Intervention types for clinical trials with mutations.

	Intervention Type	Number of Clinical Trials	Percent mapped to CT with Genes	Percent with RSid	Percent with mutations
1	Behavioral	35,450	51.5%	0.055%	0.12%
2	Biological	16,370	54.6%	0.084%	0.93%
3	Combination Product	1152	61.5%	0.11%	0.52%
4	Device	43,079	60.1%	0.025%	0.1%
5	Diagnostic Test	6,299	67.6%	0.255%	0.4%
6	Dietary Supplement	10,882	55.7%	0.24%	0.36%
7	Drug	98,048	65.9%	0.14%	**1.4%**
8	Genetic	1,189	72.8%	**2.34%**	**4.1%**
9	Other	52,885	54.8%	0.12%	0.43%
10	Procedure	33,045	62.8%	0.035%	0.27%
11	Radiation	3,650	**83.2%**	0.12%	**1.04%**

Eleven different categories of Interventions along with the number of unique tags in each category. Additionally, the percent of clinical trials that mapped to HPO nodes with associated genes, clinical trials with RSids, and clinical trials with protein mutations are illustrated.

**Fig 5:**
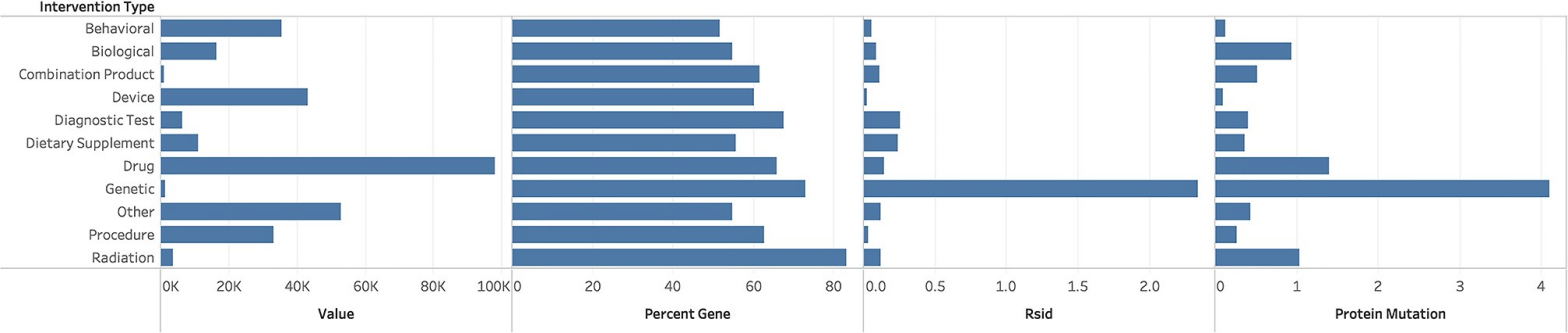
Percentage of clinical trials in each of the eleven categories with RSids and protein mutations. (a) The first graph shows the relative frequency of clinical trials in each of the eleven Intervention types. (b) The second shows the percent of clinical trials in each of the categories that link to an HPO term and has an associated gene. (c) The third shows the relative frequency of clinical trials in each of the categories that had an associated RSid. (d) The fourth shows the percent of clinical trials in each of the categories that had an associated protein mutation.

Each Intervention tag was categorized into one of two mutually-exclusive categories: one that had a clinical trial with an HPO term (and consequently was associated with a gene), and the other that did not have an HPO term. The last column shows the percentage of Intervention Types that were mapped to clinical trials with associated genes; the Radiation Intervention Type had the highest percentage with 83.2%, indicating the dependence of Radiation research on genetic information. Fig 5 shows four subgraphs: the first illustrates the relative frequency distribution of clinical trial interventions across the eleven categories; the second is the percent distribution of clinical trials with HPO nodes associated with genes; the third depicts the percent of the clinical trials which have an RSid, and the fourth displays percentages of clinical trials that have a protein mutation. As expected, clinical trials with the “Genetic Intervention” type had the highest percent of clinical trials with SNPs and protein mutations, with 2.34% and 4.1%. Intervention types “Drug” and “Radiation” also had a high incidence of protein mutations with 1.4% and 1.04%, respectively, of the clinical trials referencing mutations.

### Machine learning application: Results

Three representative HPO nodes were selected to demonstrate the results of the clustering by SNP. The HPO nodes most similar to each are shown in Table 8 and discussed below.

1. **HP:0001909 Leukemia**: As expected, the most common HPO nodes related to “HP:0001909 Leukemia” are all associated with different kinds of Leukemia, validating the methodology. Yet, lower in the list, nodes like “HP:0004757 Paroxysmal atrial fibrillation” seem out of place. However, patients with Leukemia are treated with the drug, Ibrutinib, a Bruton’s tyrosine kinase inhibitor [48] that has two adverse effects: atrial fibrillation and bleeding. Therefore, “HP:0004757 Paroxysmal atrial fibrillation” is correctly linked to “HP:0001909 Leukemia,” illustrating that this machine learning example incorporates multiple features of HPO Nodes and their corresponding mutations to highlight interesting and possibly novel correlations. Similarly, Leukemia is related to Dysmenorrhea [49] and Depressivity [50] through this methodology, illustrating the effectiveness of such Machine Learning applications in possibly finding novel correlations between diseases/conditions.
2. **HP:0000819 Diabetes mellitus**: As expected, “HP:0000819 Diabetes mellitus” is associated with different elements of diabetes, kidneys, weight, insulin, the gastrointestinal tract, livers, and the cardiovascular system, further validating the methodology and pipeline.
3. **HP:0001824 Weight loss**: As the last example, the generic non-disease term “Weight Loss” was selected. “Weight Loss” still worked outstandingly in the algorithm as common correlations were related to the gastrointestinal tract, blood-forming tissues, diabetes, kidneys, insulin, liver, and the cardiovascular system.

**Table 8:** Related HPO terms using co-occurrences of RSids and HPO terms.

	HPO Id	HPO Term	Related HPO Term	Score
1	HP:0001909	Leukemia	HP:0012324 Myeloid leukemia	0.69
			HP:0005526 Lymphoid leukemia	0.58
			HP:0005506 Chronic myelogenous leukemia	0.58
			HP:0002665 Lymphoma	0.45
			HP:0004808 Acute myeloid leukemia	0.39
			HP:0005550 Chronic lymphatic leukemia	0.37
			HP:0012539 Non-Hodgkin lymphoma	0.26
			HP:0004757 Paroxysmal atrial fibrillation	0.13
			HP:0100607 Dysmenorrhea	0.12
			HP:0000716 Depressivity	0.1
2	HP:0000819	Diabetes mellitus	HP:0005978 Type II diabetes mellitus	0.57
			HP:0100651 Type I diabetes mellitus	0.5
			HP:0000077 Abnormality of the kidney	0.45
			HP:0011998 Postprandial hyperglycemia	0.45
			HP:0012622 Chronic kidney disease	0.38
			HP:0001824 Weight loss	0.29
			HP:0001392 Abnormality of the liver	0.27
			HP:0000855 Insulin resistance	0.27
			HP:0011024 Abnormality of the gastrointestinal tract	0.25
			HP:0001871 Abnormality of blood and blood-forming tissues	0.25
			HP:0001397 Hepatic steatosis	0.24
			HP:0001513 Obesity	0.12
			HP:0001626 Abnormality of the cardiovascular system	0.067
			HP:0001677 Coronary artery atherosclerosis	0.057
3	HP:0001824	Weight loss	HP:0011024 Abnormality of the gastrointestinal tract	
			HP:0001871 Abnormality of blood and blood-forming tissues	0.58
			HP:0000819 Diabetes mellitus	0.58
			HP:0012622 Chronic kidney disease	0.29
			HP:0100651 Type I diabetes mellitus	0.29
			HP:0001513 Obesity	0.29
			HP:0000077 Abnormality of the kidney	0.26
			HP:0001392 Abnormality of the liver	0.2
			HP:0000855 Insulin resistance	0.2
			HP:0001397 Hepatic steatosis	0.18
			HP:0001626 Abnormality of the cardiovascular system	0.15

Results from finding similar HPO terms using occurrence of RSids as dimensions. The above results are representative, and the complete analysis, with the Java API, can be downloaded from the SNP Miner homepage.

Readers are encouraged to use the APIs developed to try out the complete analysis using both SNPs and protein mutations.

## Conclusion and future work

In this work, protein mutations and SNPs were successfully mined from ClinicalTrials.gov. Additionally, mutations and clinical trials were associated with HPO and MeSH ontologies. The benefits of using ontologies to help normalize free-formed text were demonstrated, and the mapping from MeSH to HPO also enabled the finding of genes associated with the HPO term. Unique reports for each mutation and clinical trial were created, helping researchers mine associations between mutations, genes, and diseases. These reports are freely available on the web, along with APIs (Java and Google Colab notebooks) for programmatic access. Further, the publicly-available site (http://snpminertrails.com) contains analysis at multiple time points, further providing researchers with longitudinal information about clinical trials and associated entities, as well as demonstrating the reproducibility of the methods. The programmatic access of the data connecting SNPs and protein mutations with MeSH and HPO terms can also be useful for machine learning, as demonstrated above.

Future work would enhance the developed framework to include other mutation types and generate further insights from ClinicalTrials.gov data. This framework, utilizing the created pipeline, can additionally be applied to other scientific corpora, such as PubMed [51] and PubMed Central [52], another area of future work. Additional insights can be obtained by extracting biomedical entities from the clinical trials corpus. For e.g., U.S. Food and Drug Administration (FDA), Center for Biologics Evaluation and Research (CBER), and Center for Drug Evaluation and Research (CDER) [53] have a rich repository of drug information.

## Supporting information

S1 Fig Screen shots of SNPMiner homepage, various reports, and API toolkts. (PDF)

S2 Fig Graphs of different analysis reports. (PDF)
