# Supplementary material for: Unique insights from ClinicalTrials.gov by mining protein mutations and RSids in addition to applying the Human Phenotype Ontology
Source: PLoS One. 2020 May 27;15(5):e0233438. doi: 10.1371/journal.pone.0233438 (PMC7252633; doi:10.1371/journal.pone.0233438)
Supplement: S1 Fig — (PDF) [file pone.0233438.s001.pdf]

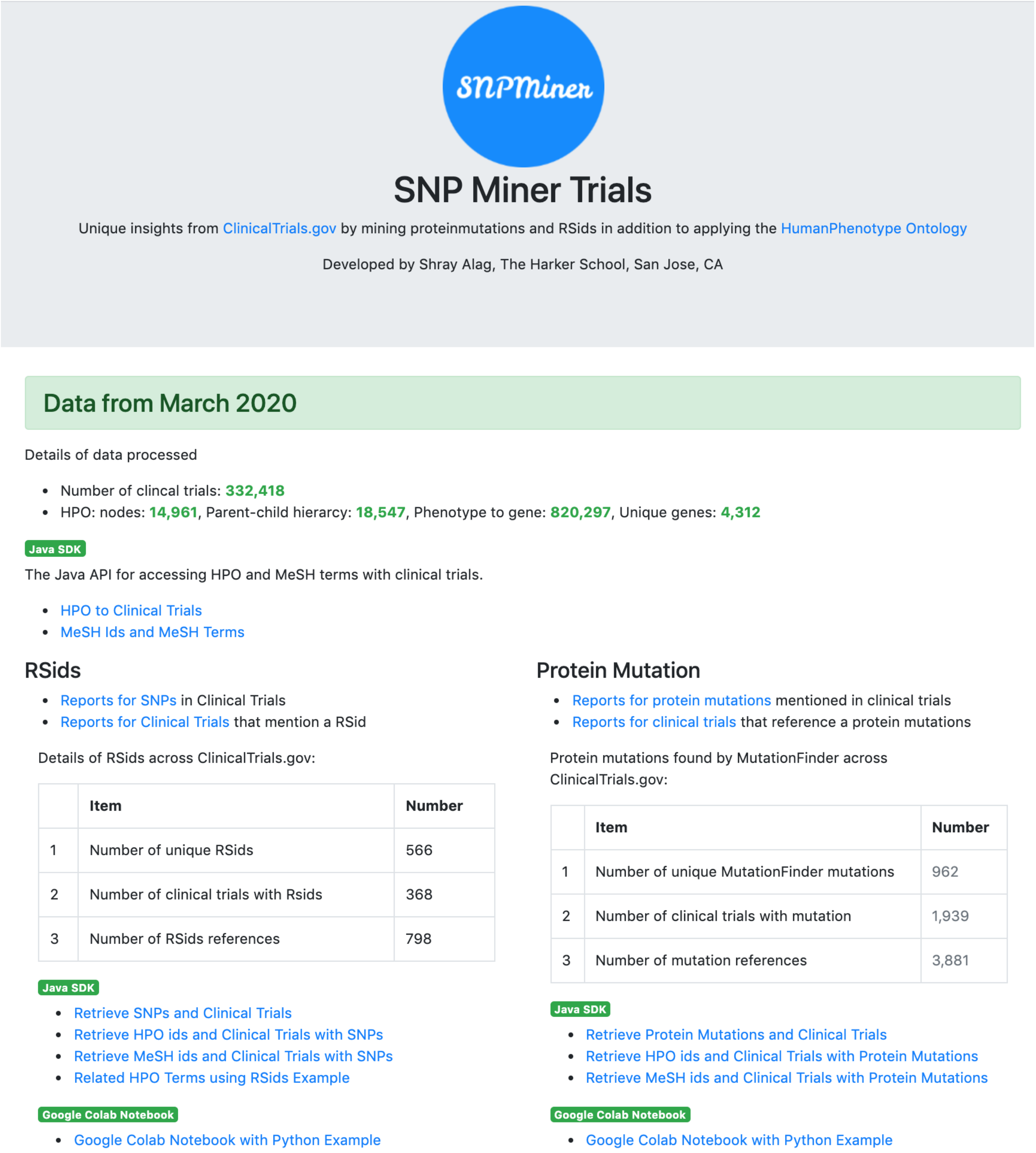

SNPMiner

SNP Miner Trials

Unique insights from ClinicalTrials.gov by mining proteinmutations and RSids in addition to applying the HumanPhenotype Ontology

Developed by Shray Alag, The Harker School, San Jose, CA

Data from March 2020

Details of data processed

- Number of clincal trials: **332,418**
- HPO: nodes: **14,961**, Parent-child hierarcy: **18,547**, Phenotype to gene: **820,297**, Unique genes: **4,312**

Java SDK

The Java API for accessing HPO and MeSH terms with clinical trials.

- HPO to Clinical Trials
- MeSH Ids and MeSH Terms

RSids

- Reports for SNPs in Clinical Trials
- Reports for Clinical Trials that mention a RSid

Details of RSids across ClinicalTrials.gov:

	Item	Number
1	Number of unique RSids	566
2	Number of clinical trials with Rsids	368
3	Number of RSids references	798

Java SDK

- Retrieve SNPs and Clinical Trials
- Retrieve HPO ids and Clinical Trials with SNPs
- Retrieve MeSH ids and Clinical Trials with SNPs
- Related HPO Terms using RSids Example

Google Colab Notebook

- Google Colab Notebook with Python Example

Protein Mutation

- Reports for protein mutations mentioned in clinical trials
- Reports for clinical trials that reference a protein mutations

Protein mutations found by MutationFinder across ClinicalTrials.gov:

	Item	Number
1	Number of unique MutationFinder mutations	962
2	Number of clinical trials with mutation	1,939
3	Number of mutation references	3,881

Java SDK

- Retrieve Protein Mutations and Clinical Trials
- Retrieve HPO ids and Clinical Trials with Protein Mutations
- Retrieve MeSH ids and Clinical Trials with Protein Mutations

Google Colab Notebook

- Google Colab Notebook with Python Example

Figure 1. Screenshot of the home page of SNP Miner Trials (http://snpminertrials.com), which provides access to analytics, reports, and APIs.

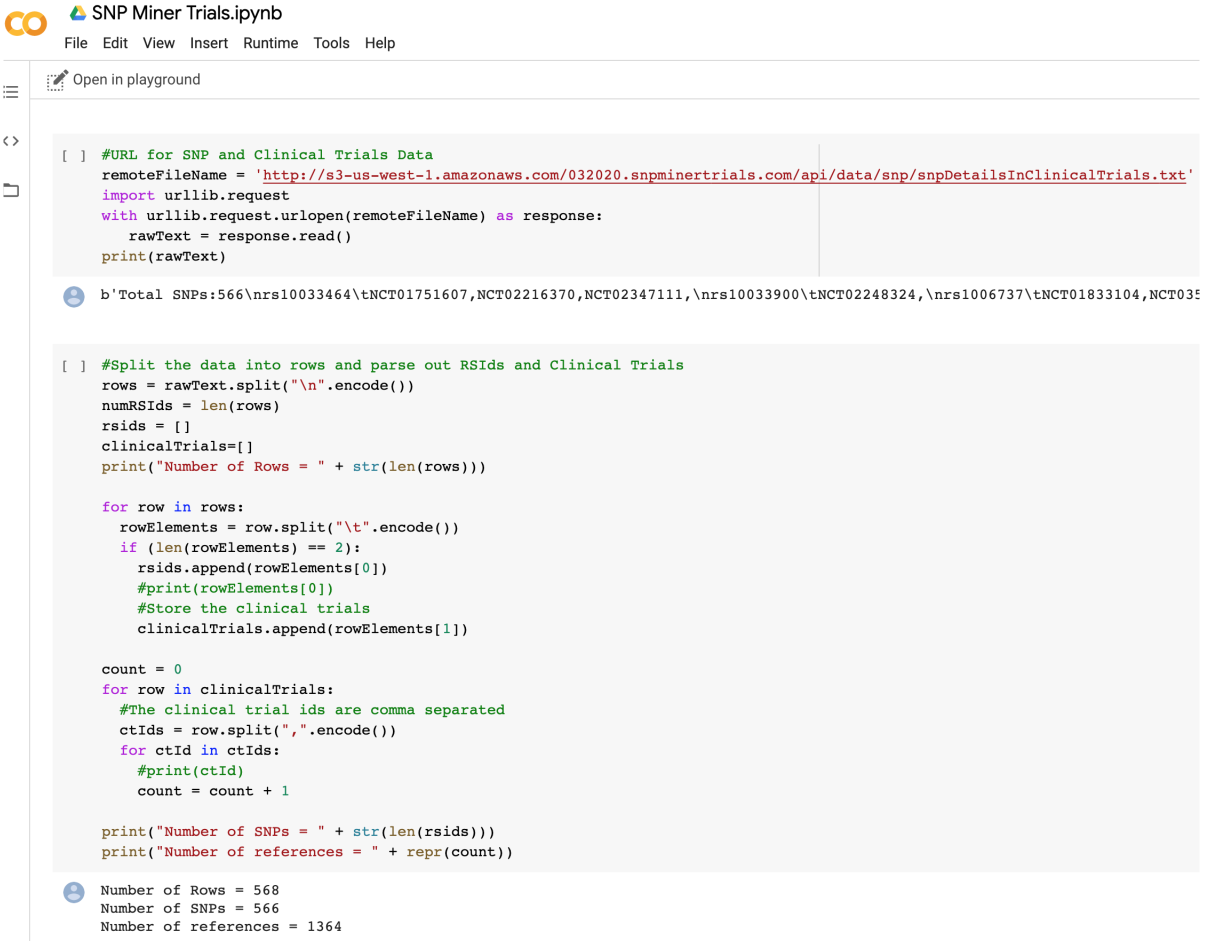

Figure 2. Screenshot of Google Colab notebook of SNP Miner Trials (http://snpminertrials.com), which provides an example Python code to access the data.

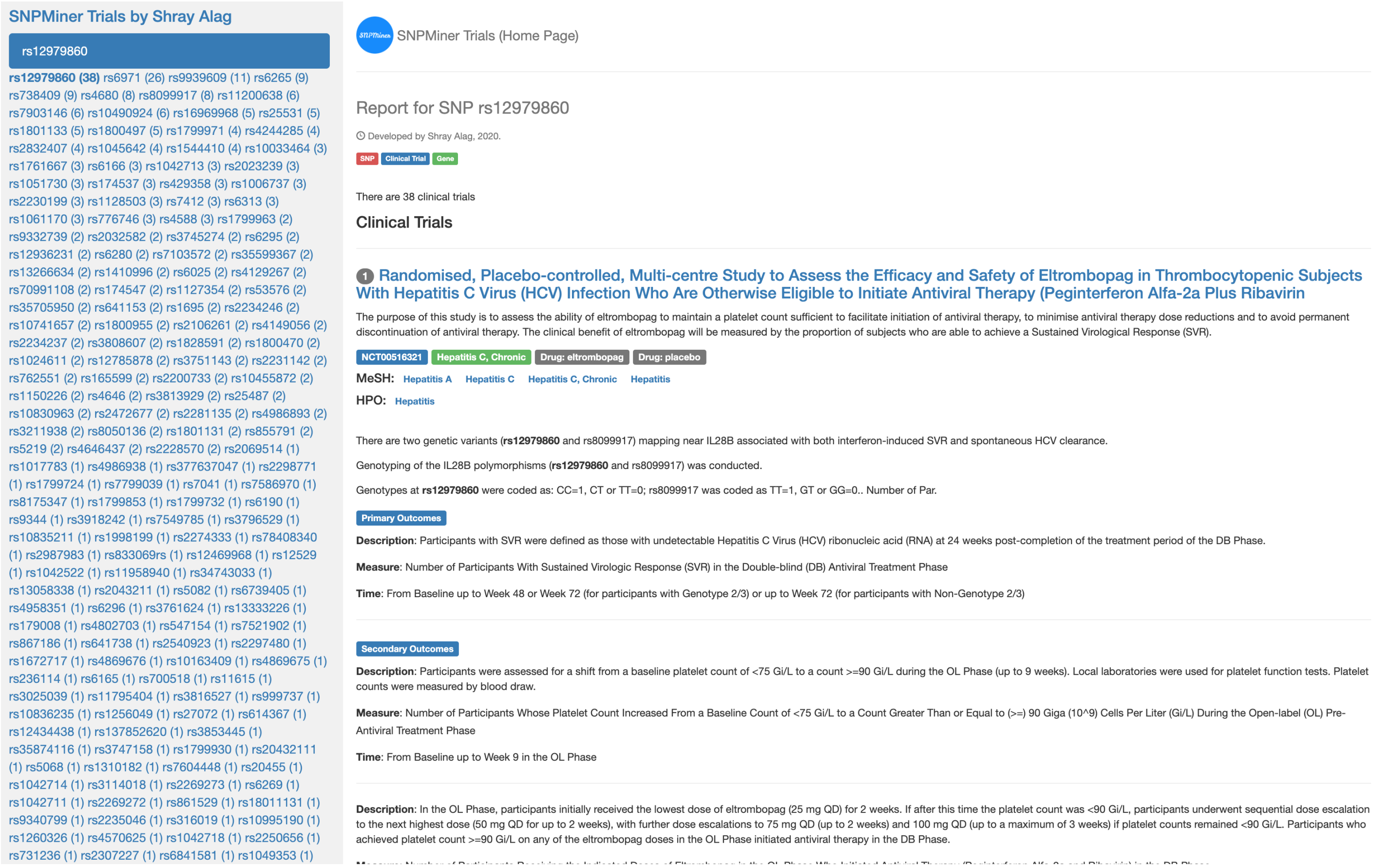

Figure 3. The top part of the report for a SNP Each SNP report contained the clinical trials in which the associated SNP was mentioned. Navigation to other SNPs was also facilitated through the left side window.

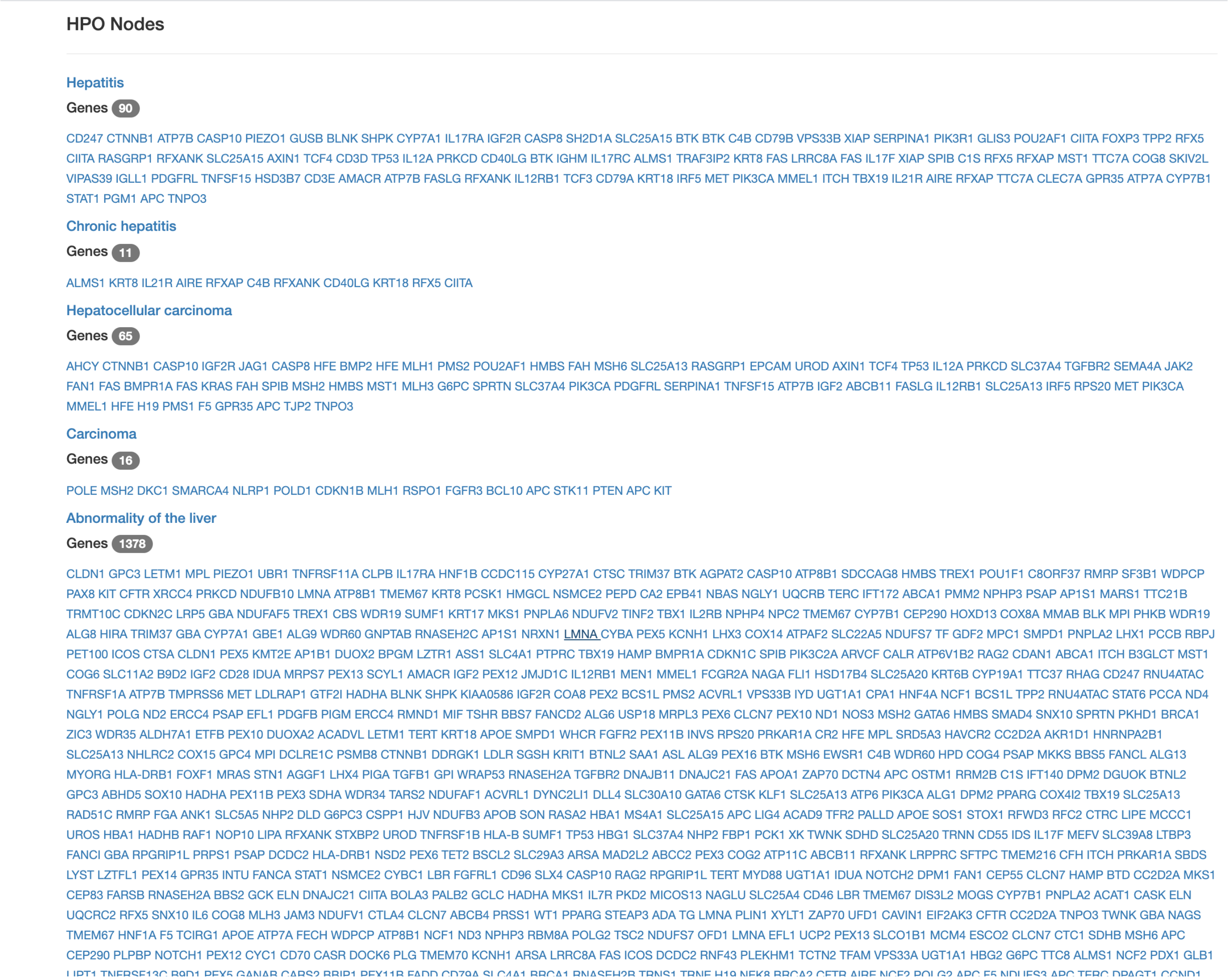

HPO Nodes

Hepatitis

Genes 90

CD247 CTNNB1 ATP7B CASP10 PIEZO1 GUSB BLNK SHPK CYP7A1 IL17RA IGF2R CASP8 SH2D1A SLC25A15 BTK BTK C4B CD79B VPS33B XIAP SERPINA1 PIK3R1 GLIS3 POU2AF1 CIITA FOXP3 TPP2 RFX5 CIITA RASGRP1 RFXANK SLC25A15 AXIN1 TCF4 CD3D TP53 IL12A PRKCD CD40LG BTK IGHM IL17RC ALMS1 TRAF3IP2 KRT8 FAS LRRC8A FAS IL17F XIAP SPIB C1S RFX5 RFXAP MST1 TTC7A COG8 SKIV2L VIPAS39 IGLL1 PDGFRL TNFSF15 HSD3B7 CD3E AMACR ATP7B FASLG RFXANK IL12RB1 TCF3 CD79A KRT18 IRF5 MET PIK3CA MMEL1 ITCH TBX19 IL21R AIRE RFXAP TTC7A CLEC7A GPR35 ATP7A CYP7B1 STAT1 PGM1 APC TNPO3

Chronic hepatitis

Genes 11

ALMS1 KRT8 IL21R AIRE RFXAP C4B RFXANK CD40LG KRT18 RFX5 CIITA

Hepatocellular carcinoma

Genes 65

AHCY CTNNB1 CASP10 IGF2R JAG1 CASP8 HFE BMP2 HFE MLH1 PMS2 POU2AF1 HMBS FAH MSH6 SLC25A13 RASGRP1 EPCAM UROD AXIN1 TCF4 TP53 IL12A PRKCD SLC37A4 TGFBR2 SEMA4A JAK2 FAN1 FAS BMPR1A FAS KRAS FAH SPIB MSH2 HMBS MST1 MLH3 G6PC SPRTN SLC37A4 PIK3CA PDGFRL SERPINA1 TNFSF15 ATP7B IGF2 ABCB11 FASLG IL12RB1 SLC25A13 IRF5 RPS20 MET PIK3CA MMEL1 HFE H19 PMS1 F5 GPR35 APC TJP2 TNPO3

Carcinoma

Genes 16

POLE MSH2 DKC1 SMARCA4 NLRP1 POLD1 CDKN1B MLH1 RSPO1 FGFR3 BCL10 APC STK11 PTEN APC KIT

Abnormality of the liver

Genes 1378

CLDN1 GPC3 LETM1 MPL PIEZO1 UBR1 TNFRSF11A CLPB IL17RA HNF1B CCDC115 CYP27A1 CTSC TRIM37 BTK AGPAT2 CASP10 ATP8B1 SDCCAG8 HMBS TREX1 POU1F1 C8ORF37 RMRP SF3B1 WDPCP PAX8 KIT CFTR XRCC4 PRKCD NDUFB10 LMNA ATP8B1 TMEM67 KRT8 PCSK1 HMGCL NSMCE2 PEPD CA2 EPB41 NBAS NGLY1 UQCRB TERC IFT172 ABCA1 PMM2 NPHP3 PSAP AP1S1 MARS1 TTC21B TRMT10C CDKN2C LRP5 GBA NDUFAF5 TREX1 CBS WDR19 SUMF1 KRT17 MKS1 PNPLA6 NDUFV2 TINF2 TBX1 IL2RB NPHP4 NPC2 TMEM67 CYP7B1 CEP290 HOXD13 COX8A MMAB BLK MPI PHKB WDR19 ALG8 HIRA TRIM37 GBA CYP7A1 GBE1 ALG9 WDR60 GNPTAB RNASEH2C AP1S1 NRXN1 LMNA CYBA PEX5 KCNH1 LHX3 COX14 ATPAF2 SLC22A5 NDUFS7 TF GDF2 MPC1 SMPD1 PNPLA2 LHX1 PCCB RBPJ PET100 ICOS CTSA CLDN1 PEX5 KMT2E AP1B1 DUOX2 BPGM LZTR1 ASS1 SLC4A1 PTPRC TBX19 HAMP BMPR1A CDKN1C SPIB PIK3C2A ARVCF CALR ATP6V1B2 RAG2 CDAN1 ABCA1 ITCH B3GLCT MST1 COG6 SLC11A2 B9D2 IGF2 CD28 IDUA MRPS7 PEX13 SCYL1 AMACR IGF2 PEX12 JMJD1C IL12RB1 MEN1 MMEL1 FCGR2A NAGA FLI1 HSD17B4 SLC25A20 KRT6B CYP19A1 TTC37 RHAG CD247 RNU4ATAC TNFRSF1A ATP7B TMPRSS6 MET LDLRAP1 GTF2I HADHA BLNK SHPK KIAA0586 IGF2R COA8 PEX2 BCS1L PMS2 ACVRL1 VPS33B IYD UGT1A1 CPA1 HNF4A NCF1 BCS1L TPP2 RNU4ATAC STAT6 PCCA ND4 NGLY1 POLG ND2 ERCC4 PSAP EFL1 PDGFB PIGM ERCC4 RMND1 MIF TSHR BBS7 FANCD2 ALG6 USP18 MRPL3 PEX6 CLCN7 PEX10 ND1 NOS3 MSH2 GATA6 HMBS SMAD4 SNX10 SPRTN PKHD1 BRCA1 ZIC3 WDR35 ALDH7A1 ETFB PEX10 DUOXA2 ACADVL LETM1 TERT KRT18 APOE SMPD1 WHCR FGFR2 PEX11B INVS RPS20 PRKAR1A CR2 HFE MPL SRD5A3 HAVCR2 CC2D2A AKR1D1 HNRNPA2B1 SLC25A13 NHLRC2 COX15 GPC4 MPI DCLRE1C PSMB8 CTNNB1 DDRGK1 LDLR SGSH KRIT1 BTNL2 SAA1 ASL ALG9 PEX16 BTK MSH6 EWSR1 C4B WDR60 HPD COG4 PSAP MKKS BBS5 FANCL ALG13 MYORG HLA-DRB1 FOXF1 MRAS STN1 AGGF1 LHX4 PIGA TGFB1 GPI WRAP53 RNASEH2A TGFBR2 DNAJB11 DNAJC21 FAS APOA1 ZAP70 DCTN4 APC OSTM1 RRM2B C1S IFT140 DPM2 DGUOK BTNL2 GPC3 ABHD5 SOX10 HADHA PEX11B PEX3 SDHA WDR34 TARS2 NDUFAF1 ACVRL1 DYNC2LI1 DLL4 SLC30A10 GATA6 CTSK KLF1 SLC25A13 ATP6 PIK3CA ALG1 DPM2 PPARG COX4I2 TBX19 SLC25A13 RAD51C RMRP FGA ANK1 SLC5A5 NHP2 DLD G6PC3 CSPP1 HJV NDUFB3 APOB SON RASA2 HBA1 MS4A1 SLC25A15 APC LIG4 ACAD9 TFR2 PALLD APOE SOS1 STOX1 RFWD3 RFC2 CTRC LIPE MCCC1 UROS HBA1 HADHB RAF1 NOP10 LIPA RFXANK STXBP2 UROD TNFRSF1B HLA-B SUMF1 TP53 HBG1 SLC37A4 NHP2 FBP1 PCK1 XK TWNK SDHD SLC25A20 TRNN CD55 IDS IL17F MEFV SLC39A8 LTBP3 FANCI GBA RPGRIP1L PRPS1 PSAP DCDC2 HLA-DRB1 NSD2 PEX6 TET2 BSCL2 SLC29A3 ARSA MAD2L2 ABCC2 PEX3 COG2 ATP11C ABCB11 RFXANK LRPPRC SFTPC TMEM216 CFH ITCH PRKAR1A SBDS LYST LZTFL1 PEX14 GPR35 INTU FANCA STAT1 NSMCE2 CYBC1 LBR FGFRL1 CD96 SLX4 CASP10 RAG2 RPGRIP1L TERT MYD88 UGT1A1 IDUA NOTCH2 DPM1 FAN1 CEP55 CLCN7 HAMP BTD CC2D2A MKS1 CEP83 FARSB RNASEH2A BBS2 GCK ELN DNAJC21 CIITA BOLA3 PALB2 GCLC HADHA MKS1 IL7R PKD2 MICOS13 NAGLU SLC25A4 CD46 LBR TMEM67 DIS3L2 MOGS CYP7B1 PNPLA2 ACAT1 CASK ELN UQCRC2 RFX5 SNX10 IL6 COG8 MLH3 JAM3 NDUFV1 CTLA4 CLCN7 ABCB4 PRSS1 WT1 PPARG STEAP3 ADA TG LMNA PLIN1 XYLT1 ZAP70 UFD1 CAVIN1 EIF2AK3 CFTR CC2D2A TNPO3 TWNK GBA NAGS TMEM67 HNF1A F5 TCIRG1 APOE ATP7A FECH WDPCP ATP8B1 NCF1 ND3 NPHP3 RBM8A POLG2 TSC2 NDUFS7 OFD1 LMNA EFL1 UCP2 PEX13 SLCO1B1 MCM4 ESCO2 CLCN7 CTC1 SDHB MSH6 APC CEP290 PLPBP NOTCH1 PEX12 CYC1 CD70 CASR DOCK6 PLG TMEM70 KCNH1 ARSA LRRC8A FAS ICOS DCDC2 RNF43 PLEKHM1 TCTN2 TFAM VPS33A UGT1A1 HBG2 G6PC TTC8 ALMS1 NCF2 PDX1 GLB1 [illegible]

Figure 4. Bottom of the report for a SNP with associated clinical trials. This report is for rs12979860 which is referenced in 38 clinical trials.

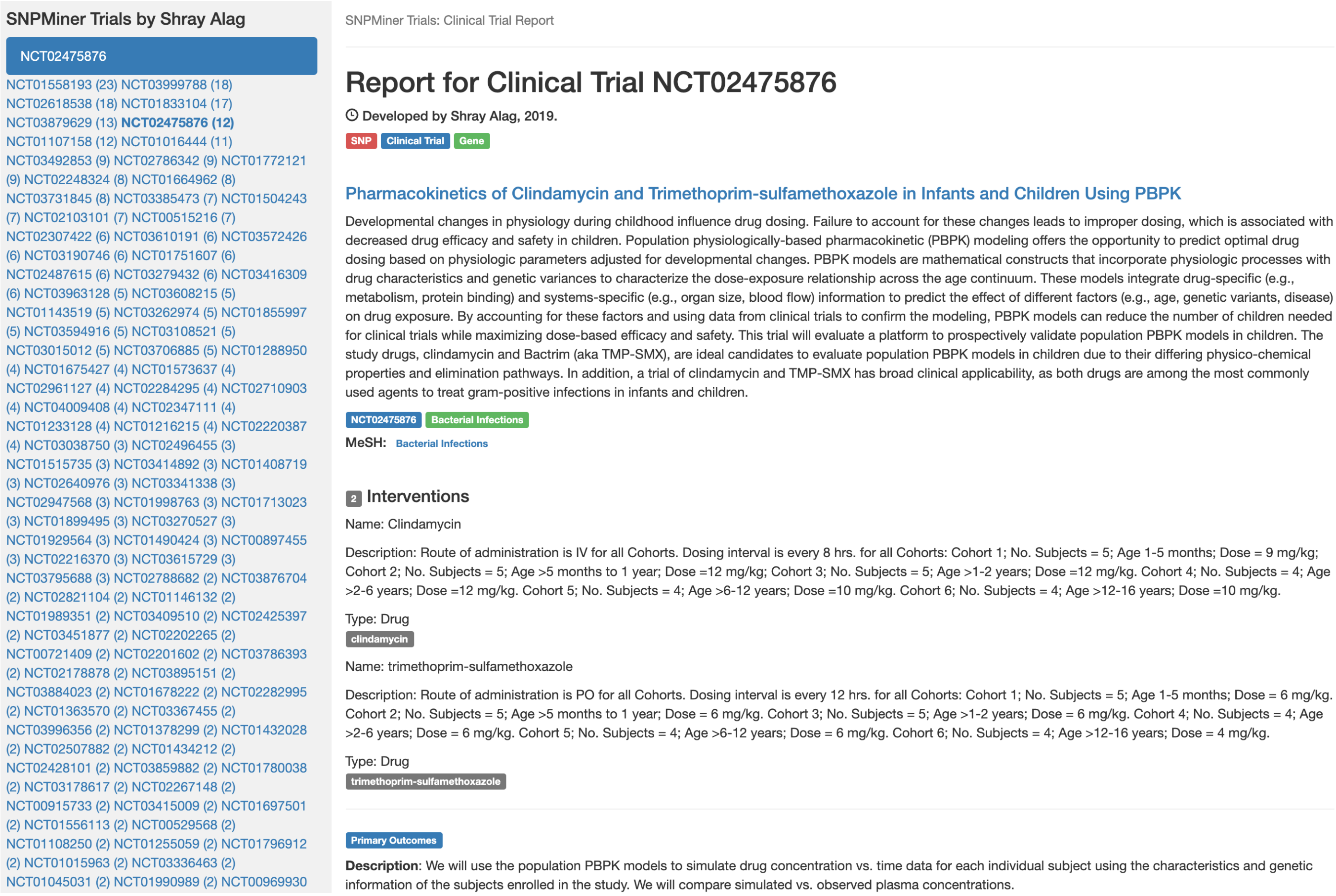

Figure 5. Report for a clinical trial with the associated RSids referenced. This report is for clinical trial NCT02475876.

There are 23 SNPs

SNPs

1 rs1042713

Adrenergic mechanisms Finally six SNPs associated with adrenergic receptors were considered: ADRA2A (adrenoceptor alpha 2A, rs553668); ADRB1 (adrenoceptor alpha B1, rs1801253); ADRB2 (adrenoceptor alpha B2, **rs1042713**; ADRB3 (adrenoceptor alpha B3, rs4994); SLC6AC (noradrenaline transporter, rs5569 and rs2242447).

2 rs1079598

Dopamine receptor D1 (DRD1, rs4532); Dopamine receptor D2 (DRD2, **rs1079598**, rs1800497); Dopamine receptor D3 (DRD3, rs6280); Dopamine receptor D4 (DRD4, rs1800955).

3 rs1150226

SNPs associated with various serotonin receptors were also examined: genetic variations of the HTR1A gene (5-HT1A receptor, rs6295); HTR1B gene (5-HT1B receptor, rs6296); HTR2A gene (5-HT2A receptor, rs6311); HTR2B gene (5-HT2B receptor, rs1549339); HTR2C gene (5-hydroxytryptamine receptor 2C, rs518147); HTR3A gene (5-hydroxytryptamine receptor 3A, **rs1150226**); HTR3B (5-HT3B receptor, rs1672717); HTR4 gene (5-HT4 receptor, rs2278392).

4 rs1549339

SNPs associated with various serotonin receptors were also examined: genetic variations of the HTR1A gene (5-HT1A receptor, rs6295); HTR1B gene (5-HT1B receptor, rs6296); HTR2A gene (5-HT2A receptor, rs6311); HTR2B gene (5-HT2B receptor, **rs1549339**); HTR2C gene (5-hydroxytryptamine receptor 2C, rs518147); HTR3A gene (5-hydroxytryptamine receptor 3A, rs1150226); HTR3B (5-HT3B receptor, rs1672717); HTR4 gene (5-HT4 receptor, rs2278392).

5 rs16111115

Dopamine The SNPs associated with the metabolism and functioning of dopamine were: Dopamine beta hydroxylase (DBH, **rs16111115**); Dopamine transporter (DAT1, rs2550946); Catechol-O-methyltransferase (COMT, rs4680, rs6269).

6 rs1672717

SNPs associated with various serotonin receptors were also examined: genetic variations of the HTR1A gene (5-HT1A receptor, rs6295); HTR1B gene (5-HT1B receptor, rs6296); HTR2A gene (5-HT2A receptor, rs6311); HTR2B gene (5-HT2B receptor, rs1549339); HTR2C gene (5-hydroxytryptamine receptor 2C, rs518147); HTR3A gene (5-hydroxytryptamine receptor 3A, rs1150226); HTR3B (5-HT3B receptor, **rs1672717**); HTR4 gene (5-HT4 receptor, rs2278392).

7 rs1800497

Dopamine receptor D1 (DRD1, rs4532); Dopamine receptor D2 (DRD2, rs1079598, **rs1800497**); Dopamine receptor D3 (DRD3, rs6280); Dopamine receptor D4 (DRD4, rs1800955).

Figure 6. SNPs within a clinical trial, with the sentences in which they appear.

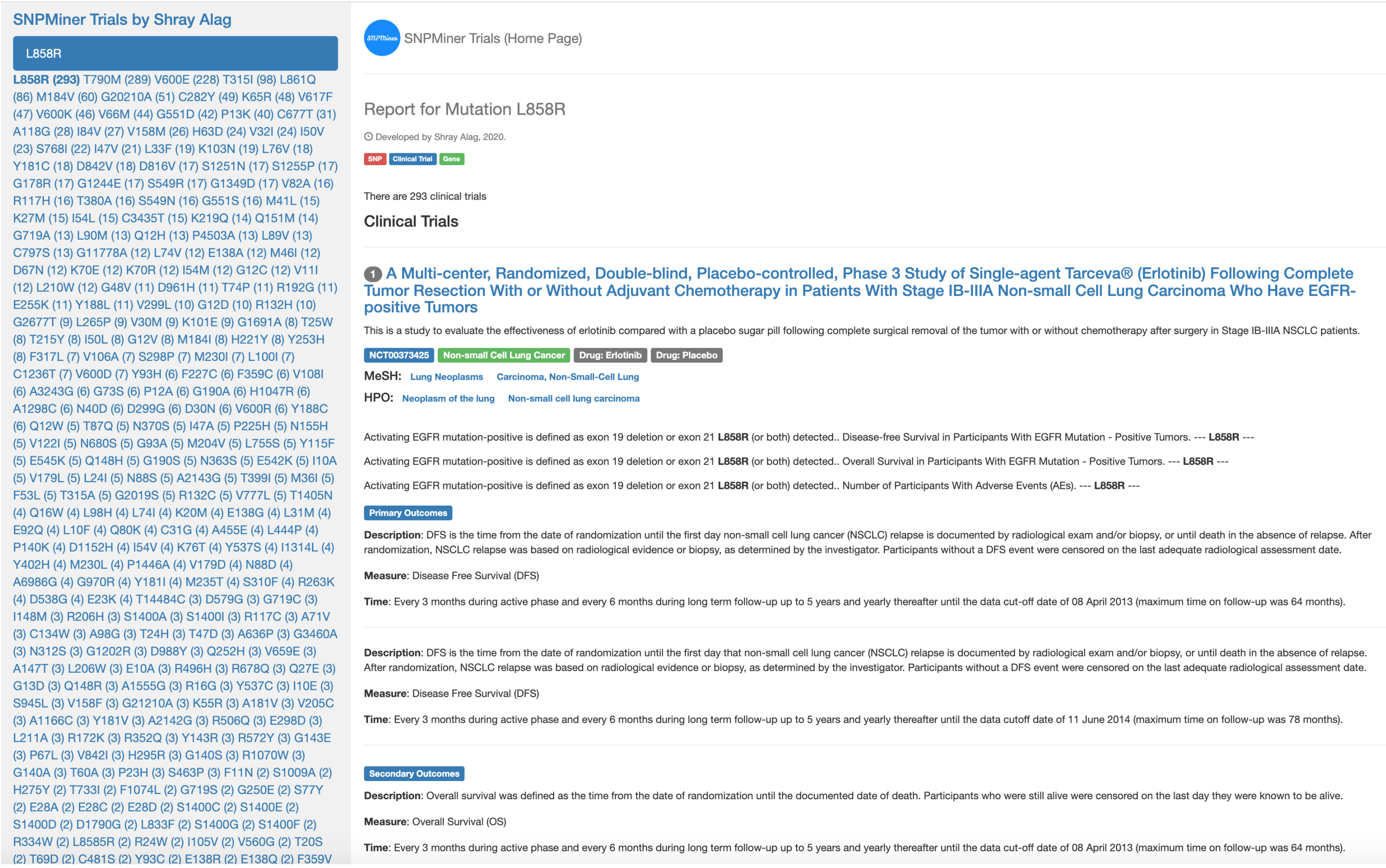

Figure 7. The upper section of the report for a protein mutation Each protein-mutation report contains the clinical trials in which the associated protein-mutation was mentioned. Navigation to other protein mutations is also facilitated through the left side window. The report is publicly-available at https://snpminertrials.com
