# Supplementary material for: Unique insights from ClinicalTrials.gov by mining protein mutations and RSids in addition to applying the Human Phenotype Ontology
Source: PLoS One. 2020 May 27;15(5):e0233438. doi: 10.1371/journal.pone.0233438 (PMC7252633; doi:10.1371/journal.pone.0233438)
Supplement: S2 Fig — (PDF) [file pone.0233438.s002.pdf]

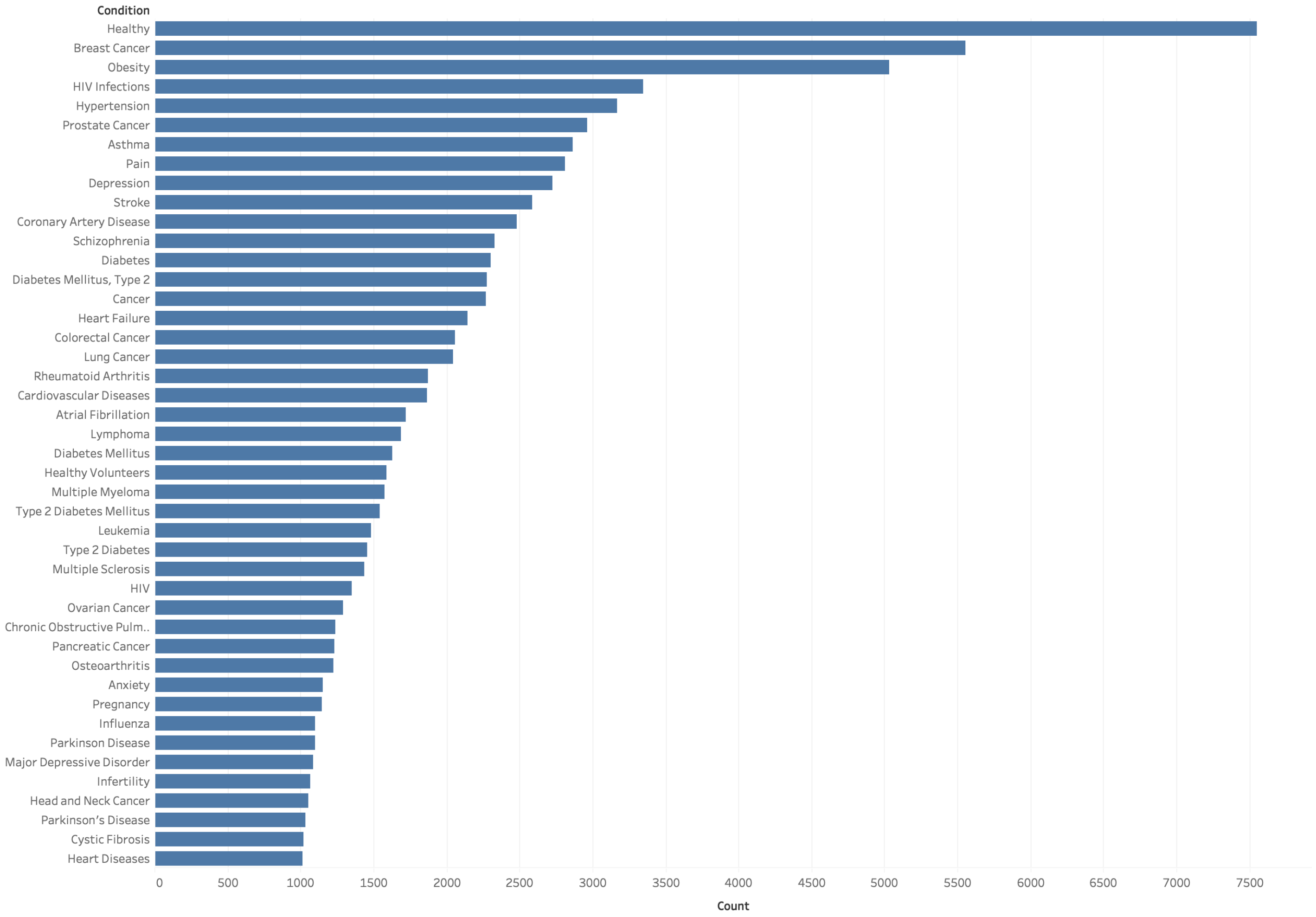

Figure 1. Frequency of different conditions across the clinical trials. These number across 332,418 clinical trials, with 87,656 unique conditions and 559,918 total condition mentions.

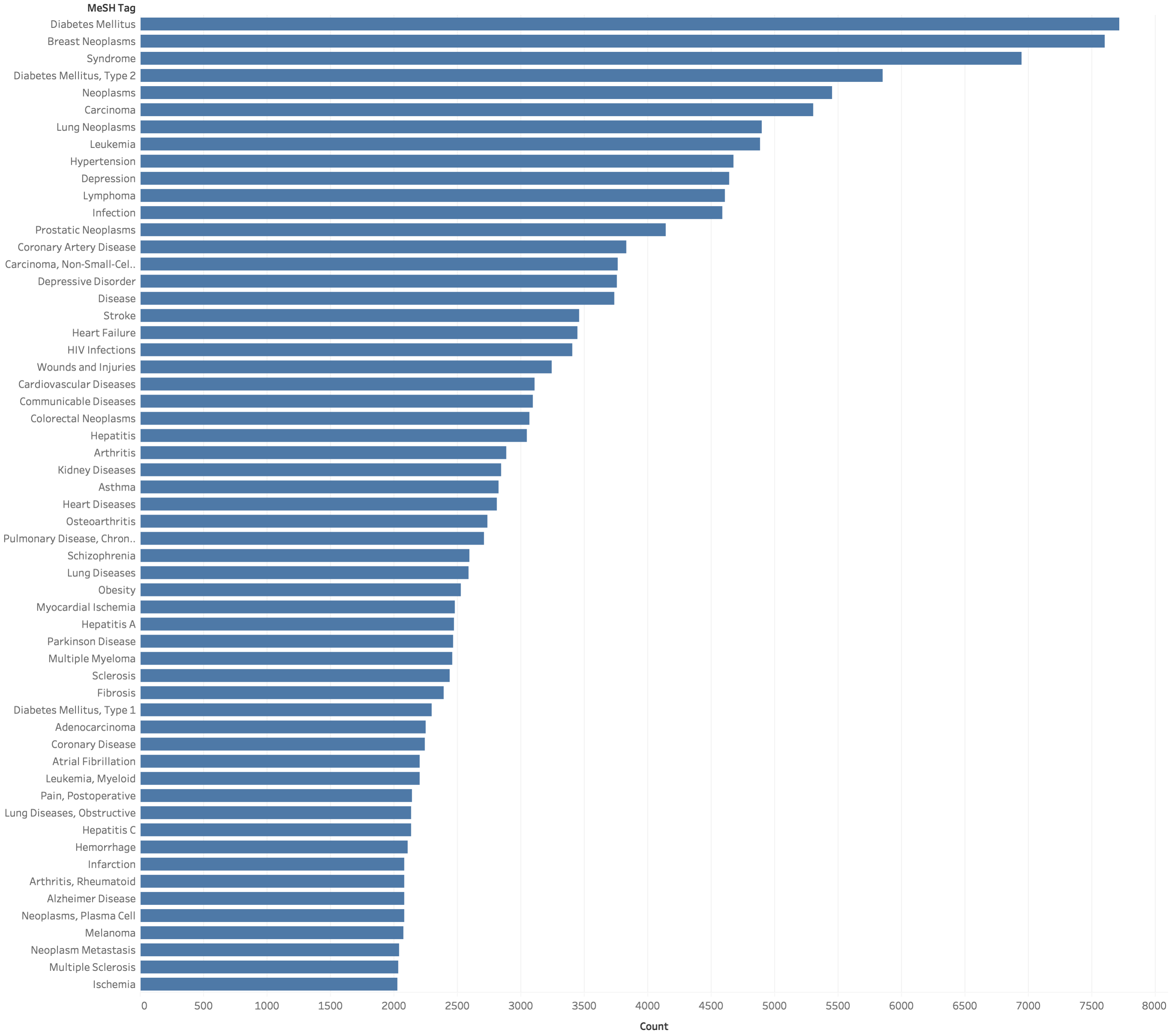

Figure 2. Frequency of different MeSH terms across clinical trials. All MeSH terms shown above are tagged by at least 2,000 documents. There are 6,643 unique MeSH terms that have been cited 568,784 times across 332,418 clinical trials.

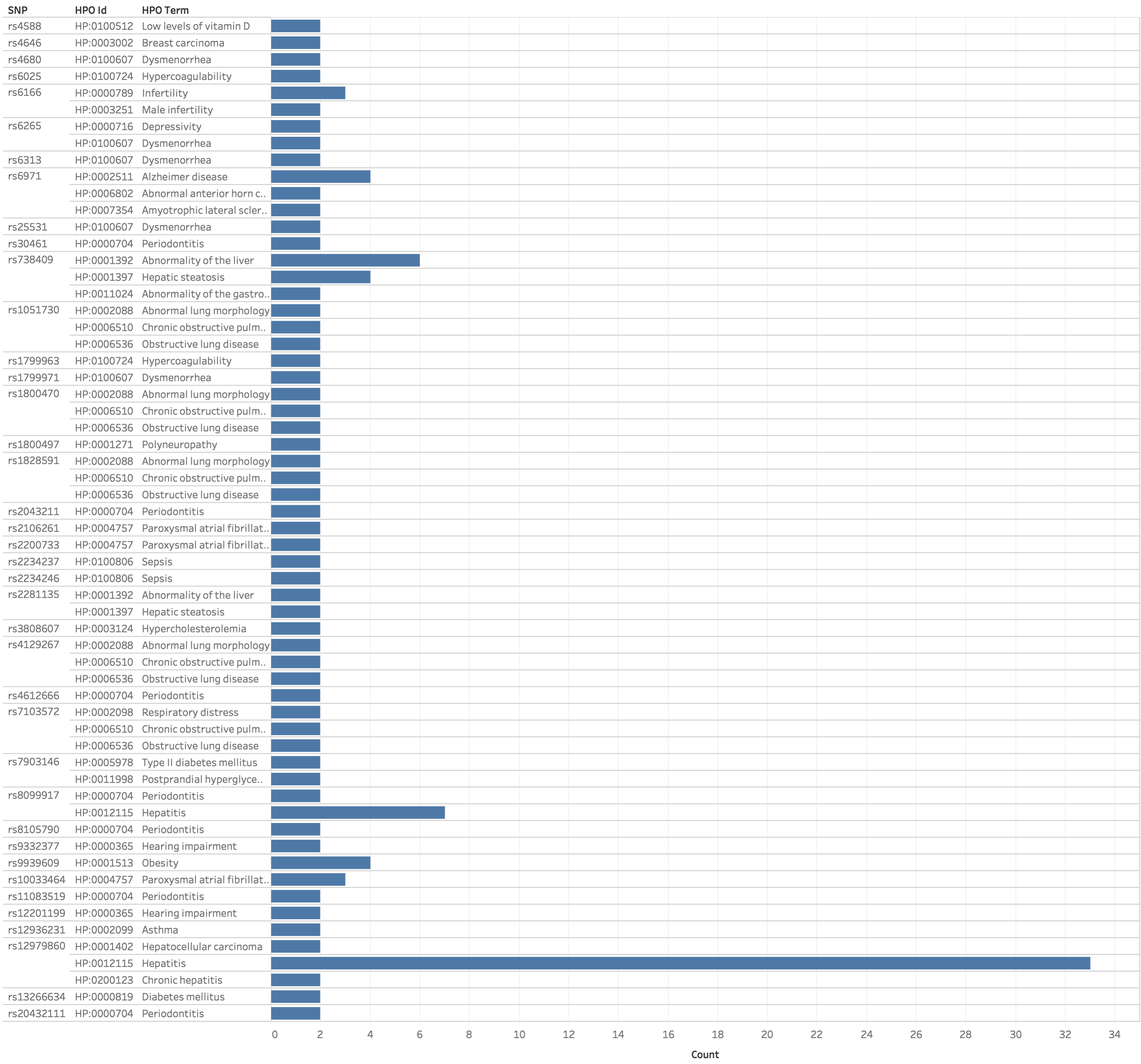

Figure 3. SNPs (denoted by RSid) with associated HPO terms and relative frequency across clinical trials with RSids.

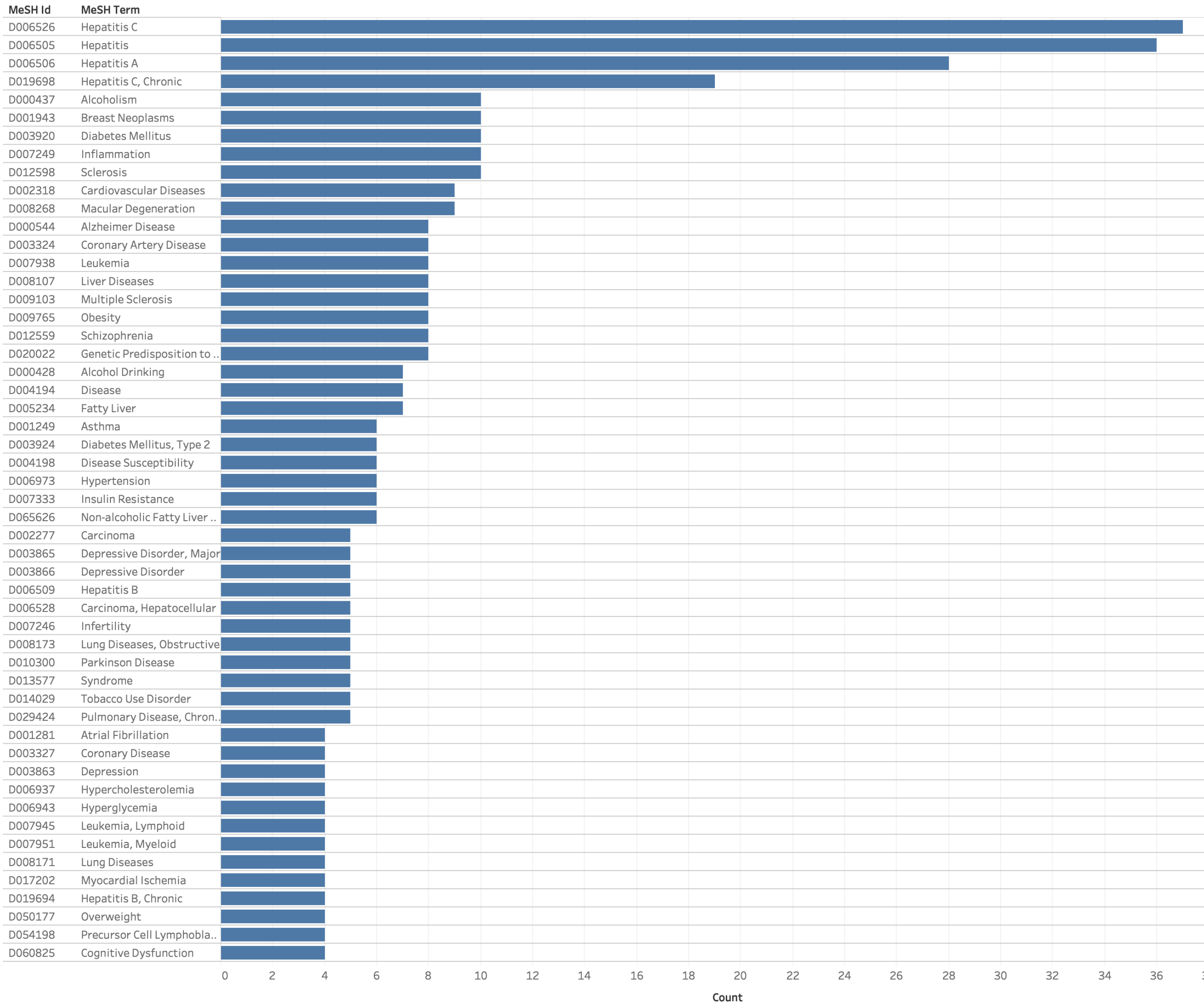

Figure 4. Relative frequency of MeSH terms used to tag clinical trials with RSids.

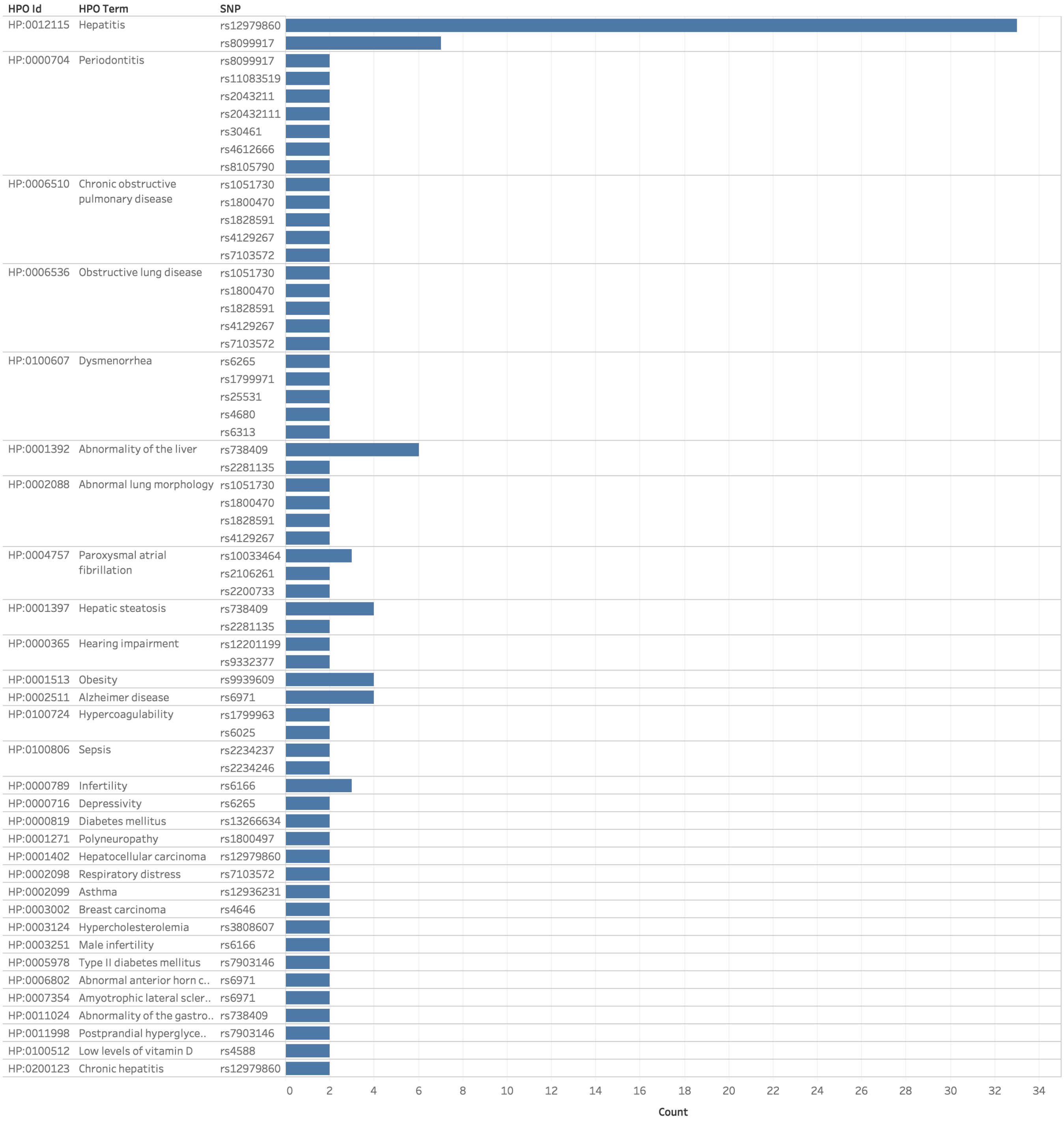

Figure 5. Relative frequency of HPO terms associated with RSid-related clinical trials.
